# Bone-Eating Worms Spread: Insights into Shallow-Water *Osedax* (Annelida, Siboglinidae) from Antarctic, Subantarctic, and Mediterranean Waters

**DOI:** 10.1371/journal.pone.0140341

**Published:** 2015-11-18

**Authors:** Sergi Taboada, Ana Riesgo, Maria Bas, Miquel A. Arnedo, Javier Cristobo, Greg W. Rouse, Conxita Avila

**Affiliations:** 1 Department of Animal Biology, Faculty of Biology, Universitat de Barcelona, Barcelona, Spain; 2 Biodiversity Research Institute (IrBIO), Faculty of Biology, Universitat de Barcelona, Barcelona, Spain; 3 Centro Oceanográfico de Gijón, Instituto Español de Oceanografía (IEO), Gijón, Spain; 4 Scripps Institution of Oceanography, La Jolla, California, United States of America; Naturhistoriska riksmuseet, SWEDEN

## Abstract

*Osedax*, commonly known as bone-eating worms, are unusual marine annelids belonging to Siboglinidae and represent a remarkable example of evolutionary adaptation to a specialized habitat, namely sunken vertebrate bones. Usually, females of these animals live anchored inside bone owing to a ramified root system from an ovisac, and obtain nutrition via symbiosis with Oceanospirillales gamma-proteobacteria. Since their discovery, 26 *Osedax* operational taxonomic units (OTUs) have been reported from a wide bathymetric range in the Pacific, the North Atlantic, and the Southern Ocean. Using experimentally deployed and naturally occurring bones we report here the presence of *Osedax deceptionensis* at very shallow-waters in Deception Island (type locality; Antarctica) and at moderate depths near South Georgia Island (Subantarctic). We present molecular evidence in a new phylogenetic analysis based on five concatenated genes (*28S* rDNA, *Histone H3*, *18S* rDNA, *16S* rDNA, and *cytochrome c oxidase I*–*COI*–), using Maximum Likelihood and Bayesian inference, supporting the placement of *O*. *deceptionensis* as a separate lineage (Clade VI) although its position still remains uncertain. This phylogenetic analysis includes a new unnamed species (*O*. ‘mediterranea’) recently discovered in the shallow-water Mediterranean Sea belonging to *Osedax* Clade I. A timeframe of the diversification of *Osedax* inferred using a Bayesian framework further suggests that *Osedax* diverged from other siboglinids during the Middle Cretaceous (ca. 108 Ma) and also indicates that the most recent common ancestor of *Osedax* extant lineages dates to the Late Cretaceous (ca. 74.8 Ma) concomitantly with large marine reptiles and teleost fishes. We also provide a phylogenetic framework that assigns newly-sequenced *Osedax* endosymbionts of *O*. *deceptionensis* and *O*. ‘mediterranea’ to ribospecies Rs1. Molecular analysis for *O*. *deceptionensis* also includes a *COI*-based haplotype network indicating that individuals from Deception Island and the South Georgia Island (ca. 1,600 km apart) are clearly the same species, confirming the well-developed dispersal capabilities reported in other congeneric taxa. In addition, we include a complete description of living features and morphological characters (including scanning and transmission electron microscopy) of *O*. *deceptionensis*, a species originally described from a single mature female, and compare it to information available for other congeneric OTUs.

## Introduction

*Osedax*, commonly known as bone-eating worms, are a remarkable example of evolutionary adaptation to subsist on vertebrate bones [1]. These unusual annelids lack a mouth and gut and generally display marked sexual dimorphism: harems of paedomorphic dwarf males are hosted within the lumen of the female’s tube [1–3]. The exception is the recently described *O*. *priapus* [4]. *Osedax* females (and also the males of *O*. *priapus*) live anchored to bones thanks to a ramified root system with an ovisac, obtaining nutrition via a unique symbiosis with Oceanospirillales gamma-proteobacteria [5–7]. These microorganisms, horizontally transmitted [7], are chemoorganoheterotrophic bacteria housed in bacteriocytes within the roots that degrade the organic compounds sequestered in the bone [5–7]. Acids produced from the roots of these animals dissolve the inorganic bone matrix, thus making available nutrients (*e*.*g*. collagen, lipids) retained in the bone [8]. A recent analysis of the genome of two of the most dominant symbionts in *Osedax* has revealed their implication in degradation of proteins, likely originating from collagenous bone matrix [9].

Since the discovery of these remarkable worms in association with a deep-water whale carcass from the Monterey Bay Canyon (NE Pacific; [1]), 26 *Osedax* operational taxonomic units (OTUs) have been reported in the Pacific, the North Atlantic, and the Southern Ocean (see [10]). Despite the bulk of taxa being described from deep waters, *Osedax* presents a wide bathymetric range (20 to down to 2,891 m; see [10]). Under a phylogenetic perspective, *Osedax* has been grouped into six different clades (I-VI) according to phylogeny and external morphology of females [11, 12]. To some extent there also have been subclades reported that correspond with geographical region rather than bathymetry or other environmental factors [10, 12].

Reproduction is one of the biological features that has attracted most studies in *Osedax*. Unlike other siboglinids, sex in *Osedax* is supposed to be environmentally determined: larvae settling on exposed bones develop as females. They secrete mucus to build a tube and develop their palps, roots, and ovisac [1, 2, 13, 14]. On the other hand, larvae landing on females become dwarf males, which retain traits of siboglinid trochophore larvae [1, 2, 14]. Fertilization in *Osedax* appears to be internal: males lying on the female’s tube inject the elongated sperm they accumulate at their head through the oviduct and it is then stored at the ovarian tissue until fertilization [3, 15]. Once fertilized, oocytes are arrested in development until after fertilization and spawning of lecithotrophic trochophore larvae, which allow these organisms dispersal and colonization of new appropriate substrates [2, 7]. This marked sexual dimorphism in *Osedax* has recently been challenged with the discovery of *O*. *priapus*, a species whose males resemble females, which also live anchored to the bone through the roots, have palps, and harbor endosymbionts in their roots. The absence of dwarf males in *O*. *priapus* represents a character reversal for *Osedax*, though some dwarf male traits are retained by the males [4].

Among the more recently studied *Osedax* are those living in the Southern Ocean, which accounts for five species that have recently been described: *O*. *antarcticus*, *O*. *crouchi*, *O*. *rogersi*, and *O*. *nordenskjoeldi*, inhabiting waters deeper than 500 m, and *O*. *deceptionensis*, a species described from shallow-waters [10, 12]. Phylogenetically, the deep-water Antarctic *Osedax* cluster together in Clade II with other *Osedax* nude-palp forms, while *O*. *deceptionensis*, although with a still unresolved position, appears to be placed as the only species in Clade VI [10], or as part of the nudepalp Clade II [4].

The study here assesses again the phylogenetic position of *O*. *deceptionensis* using a dataset based on five genes instead of the three genes that were previously available. In addition, our phylogenetic analysis includes a new unnamed species recently discovered in the shallow-water Mediterranean Sea (hereafter *Osedax* ‘mediterranea’). A Bayesian molecular clock approach is further used to investigate the origins and diversification of *Osedax*. We also place newly-sequenced *Osedax* endosymbionts of *O*. *deceptionensis* and *O*. ‘mediterranea’ in a phylogenetic framework. The molecular information of *O*. *deceptionensis* is supplemented by an haplotype network including 18 organisms from Deception Island–Antarctica–(type locality) and two additional organisms collected from the South Georgia Island–Subantarctic–. Our study also provides a complete description of living biological features and morphological characters (including scanning and transmission electron microscopy) of *O*. *deceptionensis*, a species that was originally described from a single mature female.

## Materials and Methods

### Sample collection and preservation

**Fig 1:**
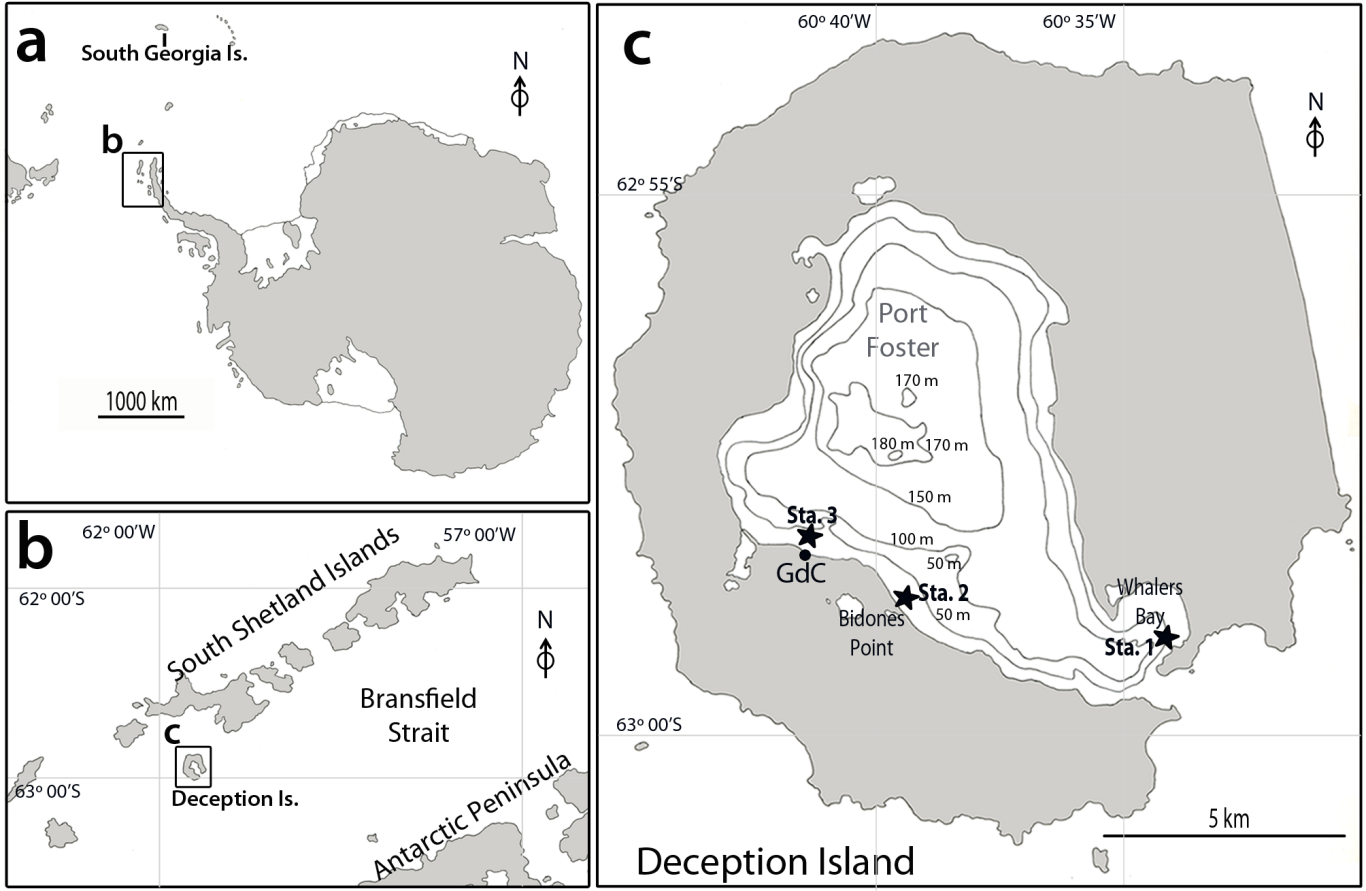
a Map of Antarctica showing the areas where samples of *Osedax deceptionensis* were collected: South Shetland Islands (*b*) and South Georgia Island, b South Shetland Islands area showing location of Deception Island, c Deception Island. GdC, *Gabriel de Castilla* Spanish Antarctic Base; Sta. 1, station at Whalers Bay; Sta. 2, station at Bidones Point; Sta. 3, station in front of the GdC Spanish Antarctic Base.

Bones for the experiments in Antarctic waters were obtained from a caudal fin of a common minke whale (*Balaenoptera acutorostrata*) dead-stranded in Andalucía (SW Spain; 36° 31' 41.39" N 6°18' 44.15" W) in 2012. The use of bones for experimental purposes was authorized by the Consejería de Medio Ambiente from the Junta de Andalucía, Spain. Bones were de-fleshed, cut into four pieces and drilled in order to ease their further attachment to experimental moorings. After this, bones were frozen until deployment via SCUBA-diving at ca. 10 m depth on the seabed of Port Foster, Deception Island (South Shetland Islands, Antarctica) at three different sites (Whalers Bay, Bidones Point, and *Gabriel de Castilla* Antarctic Spanish Base; Fig 1C, Sta. 1–3; Table 1), 8–9 January 2012. Bones were attached to pieces of ballast to avoid displacement from their original position. In order to protect bones and their associated fauna from common predators present in the area (*e*.*g*., *Parborlasia corrugatus*, *Odontaster validus*), some bones at Whalers Bay and in front of the Spanish Antarctic Base were deployed inside wire cages. All bones were recovered SCUBA-diving during January 2013, after approximately a year of deployment (Table 1). After retrieval, bones were brought to the laboratory at the *Gabriel de Castilla* Spanish Antarctic Base (Deception Island) (Fig 1C, *GdC*), where they were placed into separate containers with filtered seawater (0.22-μm) and supplementary oxygenation, and kept at ambient temperature (0–5°C) during a maximum of 17 days. Bones were observed thoroughly under a stereomicroscope to investigate the occurrence of *Osedax*, resulting in bones colonized by *O*. *deceptionensis* only at Whalers Bay (Fig 1C, Sta. 1). Prior to preservation, organisms were anesthetized in a 7% solution of MgCl_2_ in fresh water, observed *in vivo*, and photographed using an Olympus C-90 compact camera. Additional material of *O*. *deceptionensis* (2 specimens) was collected in the vicinity of South Georgia Island attached to a seal bone collected via trawling at 156 m of depth by the RVIB *Nathaniel B*. *Palmer* (Table 1) and placed in 96% ethanol. In both cases, preservation of *Osedax* organisms was allowed by specific permits of the Spanish Ministry of Science and Innovation (CPE-EIA-2011-7).

**Table 1:** Summary of the Antarctic and Subantarctic bones analyzed during this study.

Site	Bone type	N Bones^a^	Latitude	Longitude	Depth (m)	Deployment		Collection	
						Temp. (°C)	Date	Temp. (°C)	Date
**Deception Island (Antarctica)**									
**Spanish Antarctic Base**	minke whale	4 + 4*	62°58' 29.3'' S	60°40' 30.3'' W	10	2	8-Jan-2012	1.5	12-Jan-2013
**Bidones Point**	minke whale	4	62°58' 42.3'' S	60°39' 06.0'' W	10	0.5	9-Jan-2012	1.7	15-Jan-2013
**Whalers Bay**	minke whale	4 (1) + 8* (5)	62°59' 23.7'' S	60°33' 41.0'' W	15	0	9-Jan-2012	1.5	12-Jan-2013
**South Georgia Island (Subantarctica)**	seal	1 (1)	54°17' 48.3'' S	37°54' 16.2'' W	156	–	–	–	18-Apr-13

*Bones inside wire cages.

^a^ In brackets, number of bones colonized by *Osedax deceptionensis*.

Also, a single individual of *Osedax* collected from the Mediterranean Sea (*O*. ‘mediterranea’) was found in association with a minke whale bone experimentally deployed at 53 m at the head of the Blanes submarine canyon (NW Mediterranean; 41°40' 15.5" N 2°53' 23.28" E). The mature female found was retracted into its elongated tube inside the bone and presented a very bad condition, although greenish roots and oocytes were clearly observed. Preservation of this *Osedax* organism was allowed by specific permits of the Spanish Ministry of Science and Innovation (CPE-EIA-2011-7)

None of the species involved in the study are endangered or protected.

Specimens are deposited at the Centre of Biodiversity Resources (CRBA, formerly Museum of Zoology) in the Faculty of Biology, Universitat de Barcelona (UB), and the Scripps Institution of Oceanography Benthic Invertebrate Collection (SIO-BIC).

### Morphological analysis

*Osedax deceptionensis* organisms collected at Deception Island were preserved for light microscopy and scanning and transmission electron microscopy (SEM and TEM, respectively). Specimens for light microscopy observations were preserved in 10% formalin buffered in seawater and transferred after 48 h to 70% ethanol. Specimens for SEM and TEM were prefixed in a solution of 2.5% glutaraldehyde in 0.4 M phosphate saline (PBS) and 0.6 M NaCl for 24 h at 4°C. Samples were then rinsed with PBS for 40 min, post-fixed in 1% osmium tetroxide-potassium ferrocyanide in PBS for 1 h, rinsed in PBS and distilled water for 1 h, and preserved in 70% ethanol. SEM samples (entire females and tubes with larvae) were dehydrated in a graded series of alcohol, critical point dried, mounted, gold-coated and imaged using a JSM-7100F Field Emission SEM at the Scientific and Technological Centers, Universitat de Barcelona (CCiT-UB). TEM samples (roots and tubes with larvae) were dehydrated in a graded series of alcohol and embedded in Spurr’s resin. Ultrathin sections obtained with an Ultracut Reichert-Jung ultramicrotome were mounted on gold grids, stained with 2% uranyl acetate for 30 min, followed by lead citrate for 10 min, an imaged using a JEM-1010 Electron Microscope at the CCiT-UB.

### DNA extraction and amplification

Organisms for DNA sequencing were preserved in 96% ethanol and stored at -20°C until sample processing. Total DNA was extracted using the REDExtract-N-Amp kit (Sigma-Aldrich, http://www.sigma.com) from a small portion of females of *O*. *deceptionensis* collected at Deception Island and from *O*. ‘mediterranea’ collected in Blanes, following the manufacturer’s instructions. The two *O*. *deceptionensis* specimens collected from the vicinities of the South Georgia Island were extracted using the DNeasy Tissue and Blood extraction kit (Qiagen, Valencia, CA, USA). About 800 bp of *28S* rDNA (*28S*; [16]), 360 bp of *Histone H3* (*H3*; [17]), 1,800 bp of *18S* rDNA (*18S*; [18]), 300 bp of *16S* rDNA (*16S*; [19]), and 1,100 bp of *cytochrome c oxidase I* (*COI*; [20]) were amplified for *O*. ‘mediterranea’. For *O*. *deceptionensis* about 750 bp of *28S*, 300 bp of *H3*, 1,700 bp of *18S*, and 1,100 bp of *COI* were amplified for phylogenetic analysis, and ca. 450 bp of *COI* [21] were amplified for demographic analysis (see below). PCR mixtures and temperature profiles are indicated in S1 Table. PCR products were purified using microCLEAN (Microzone Limited) and sequenced at the CCiT-UB on an ABI 3730XL DNA Analyser (Applied Biosystems) or at Eurofin (Alabama).

Molecular information about endosymbionts harbored in the roots of *O*. *deceptionensis* and *O*. ‘mediterranea’ confirmed the occurrence of Oceanospirillales ribotypes in both OTUs. About 780-bp of the *16S* gene was generated using Oceanospirillales-specific primers (*435F* and *1213R*; [22]). PCR mixtures and temperature profiles are indicated in S1 Table. Sequencing was done at the CCiT-UB as described above.

### Phylogenetic analysis

Molecular phylogenetic analyses in *Osedax* were conducted using datasets for *16S*, *18S*, *28S*, *H3*, and *COI* genes (S2 Table). In total, 34 terminal taxa were used in the analysis including 27 *Osedax* OTUs, 6 non-*Osedax* siboglinids and the cirratulid *Cirratulus cirratus* as outgroup for tree rooting. Overlapping sequence fragments were assembled into consensus sequences using the software Geneious vs. 6 [23], and aligned using Q-INS-I option of MAFFT [24]. The most appropriate evolutionary model for each gene (SYM+I+G for *H3* and GTR+I+G for the rest of genes) was obtained by running the alignments in jModelTest [25] via the Akaike Information Criterion (AIC). Sequences of the five genes were then concatenated and analyses were conducted under two conditions: (i) entire dataset; and (ii) reduced dataset after removing uncertain alignment positions of the sequences using Gblocks [26]. Gblocks was run using the following settings: minimum number of sequences for a flank position = 18; maximum number of contiguous non-conserved positions = 10; minimum length of a block = 5; allowed gap positions = with half.

A combined analysis using the five concatenated genes (entire dataset and Gblocked) was conducted using Maximum Likelihood analyses (ML) with RAxML [27, 28] and Bayesian inference analyses (BI) with MrBayes 3.1.2 [29]. ML were run using 10 heuristic searches (SPR and NNI) and robustness of the nodes was determined with 10 runs and 500 replicates using the GTR+I+G evolutionary model; concatenated sequences were partitioned by gene and protein coding genes (*H3* and *COI*) were partitioned into codon positions. BI analyses were run five times for each dataset with four chains for 10 million generations (2.5 million trees discarded as burn-in) sampling a tree every 1,000 generations; partition codons were used for *H3* and *COI* and the best evolutionary models previously inferred for every gene were applied. Results were visualized in FigTree v.1.4.2 [30]. In addition, ML and BI analyses were run for each gene separately under the same conditions reported above.

Minimum genetic distances based on uncorrected *p-*distance and Kimura 2 parameters (K2p) models using MEGA vs. 5.05 [31] were calculated between *O*. *deceptionensis* and *O*. ‘mediterranea’ with respect to the rest of *Osedax*. These distances were calculated using the *COI* alignment used in the phylogenetic analyses.

As for *Osedax* endosymbionts, phylogenetic analyses were conducted using newly-generated *16S* Oceanospirillales-specific fragment obtained from *O*. *deceptionensis* and *O*. ‘mediterranea’ combined with sequences of other Oceanospirillales found in [32] and the recently described Oceanospirillales of *O*. *priapus* [4], including the bacteria used as outgroups in [6]. Alignment of sequences was done as outlined above and the most appropriate evolutionary model (SYM+I+G) was obtained by running the alignment in jModelTest [25] via the Akaike Information Criterion (AIC). Phylogenetic analyses included BI analysis and were run under the same conditions described above. Following [32], we assign the term ‘ribotype’ to a distinct *16S* sequence of a bacterial strain and ‘ribospecies’ to a group of sequences that share ≥ 97% sequence similarity.

### Divergence time estimation

A time-calibrated phylogeny based on the Gblocked concatenated BI analysis was estimated by incorporating fossil calibration points and informed priors on the substitution rates, using the software BEAST v. 1.8.1 [33]. Analyses were conducted assuming unlinked, lognormal relaxed clocks for each gene and a Yule tree prior.

Estimation of divergence time in *Osedax* and in annelids in general, has been traditionally hampered by the lack of fossil calibration points. Former estimates of the timing of diversification of *Osedax* [11] were based on assuming substitution rates for the *COI* calculated either for deep-sea hydrothermal vent annelids (r = 0.21% per linage per Ma; [34]) or for shallow-water marine invertebrates isolated after the emergence of the Isthmus of Panama (r = 0.7%; [35]). Here, for the first time, we incorporate fossil calibration points to estimate the timeframe for the diversification of *Osedax*. The oldest evidence of an *Osedax*-eaten bone dated at 100–93.9 Ma [36] and was incorporated as a minimum bound for the *Osedax* stem lineage, since the marks could not be assigned to any extant lineage for obvious reasons. Similarly, the oldest evidence of a Vestimentifera, dated at ~91 Ma [37] was assigned as a minimum bound for the stem lineage leading to Vestimentifera. Incidentally, unconstrained preliminary BEAST analyses recovered a paraphyletic Vestimentifera, which also included *Sclerolinum*. Based on additional analyses and the results of recent mitogenomic studies [38], we conducted final analyses with the Vestimentifera monophyly enforced. Fossil calibrations were incorporated as an exponential distribution prior, assuming that the divergence event occurred above the minimum date (*i*.*e*., distribution offset) and declines according to an exponential distribution, such that 95% of the posterior density falls within the range [x—x + 10%] [39]. The tree root was assigned an upper bound of 518 Ma, based on the earliest fossil evidence of a polychaete [40]. The newly found *Osedax* fossil from the Mediterranean [41] provided an additional calibration point by assuming that this fossil is related to the present day Mediterranean *Osedax*. This information was included as a uniform distribution with lower bound 2.8 Ma and upper bound corresponding to the early evidence of *Osedax*.

In addition, an informed prior was incorporated for the substitution rate of the *COI* gene. A normal distribution with mean 0.0023 and standard deviation 0.005 (truncated at 0.0001) was defined for the ucld.mean parameter of the *COI* relaxed lognormal clock. These parameter values were selected to include within the prior distribution the substitution rates used in previous time divergence analyses of *Osedax*, namely 0.7 per lineage/million years and 0.23 per lineage/million years (see [11]). Diffuse uniform distributions were assigned to the remaining genes to reduce computation time (value = 0.002, upper = 0.02, lower = 0.00001). A tree including all the time constraints was obtained with the help of the program STARTTREE (http://bodegaphylo.wikispot.org/starttree_program) and included in the BEAST analyses as a starting tree.

Three independent chains were run for 50 million generations. Convergence among chains, correct mixing within chains (*i*.*e*., ESS values) and the number of burnin generations were monitored with the program TRACER 1.6 [42]. The 10% of the first generation of each chain was removed as burnin and the remaining values were combined into a single file using LogCombiner [33] and consensus trees were obtained with TreeAnnotator [33].

### Population genetic analysis

Sequences of *COI* [21] of about 450 bp from n = 20 *O*. *deceptionensis* females (n = 18 from Deception Island, n = 2 from South Georgia Island; S3 Table) were used to construct unrooted networks with the program Network vs. 4.5.1.0 (http://www.fluxus-engineering.com/sharenet.htm). The number of haplotypes, private haplotypes, and haplotype diversity were obtained with DnaSP vs. 5.10.1 [43]. Distribution of pairwise haplotype differences (mismatch distribution) was calculated using DNAsp and compared to expected frequencies assuming population size changes. Genetic divergence within *O*. *deceptionensis* was calculated based on uncorrected *p-*distance and K2p models using MEGA vs. 5.05 [31].

## Results

### Systematics

Siboglinidae Caullery, 1914

*Osedax* Rouse, Goffredi & Vrijenhoek, 2004

*Osedax deceptionensis* Taboada, Cristobo, Avila, Wiklund and Glover, 2013

(Figs 2–6)

**Fig 2:**
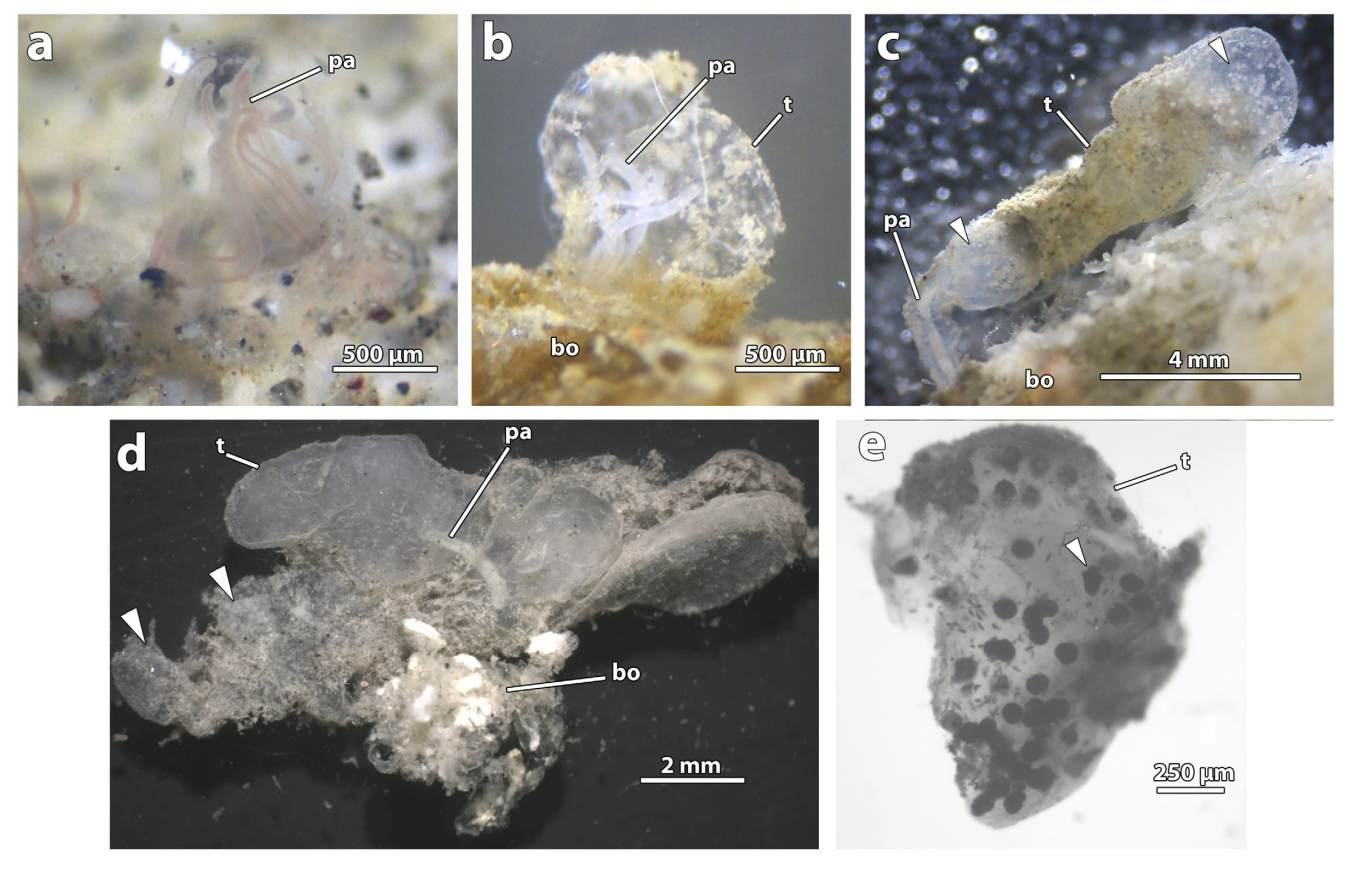
***Osedax deceptionensis*** **a** Living specimen showing translucent palps (*pa*) with red margin due to blood vessels, **b** Living specimen with palps (*pa*) retracted into a hemispherical tube (*t*) attached to the bone (*bo*), **c** Living specimen with palps (*pa*) retracted into an elongated tube (*t*) attached to the bone (*bo*). Arrows pointing areas with concentration of embryos/larvae, **d** Preserved specimen (CRBA-33236) with palps (*pa*) retracted into a multilobulated tube (*t*). Tube attached to a piece of bone (*bo*). Arrows pointing areas with concentration of embryos/larvae, **e** Piece of preserved tube (*t*) under the light microscope showing embryos/larvae. Trochophore-like larva arrowed.

**Fig 3:**
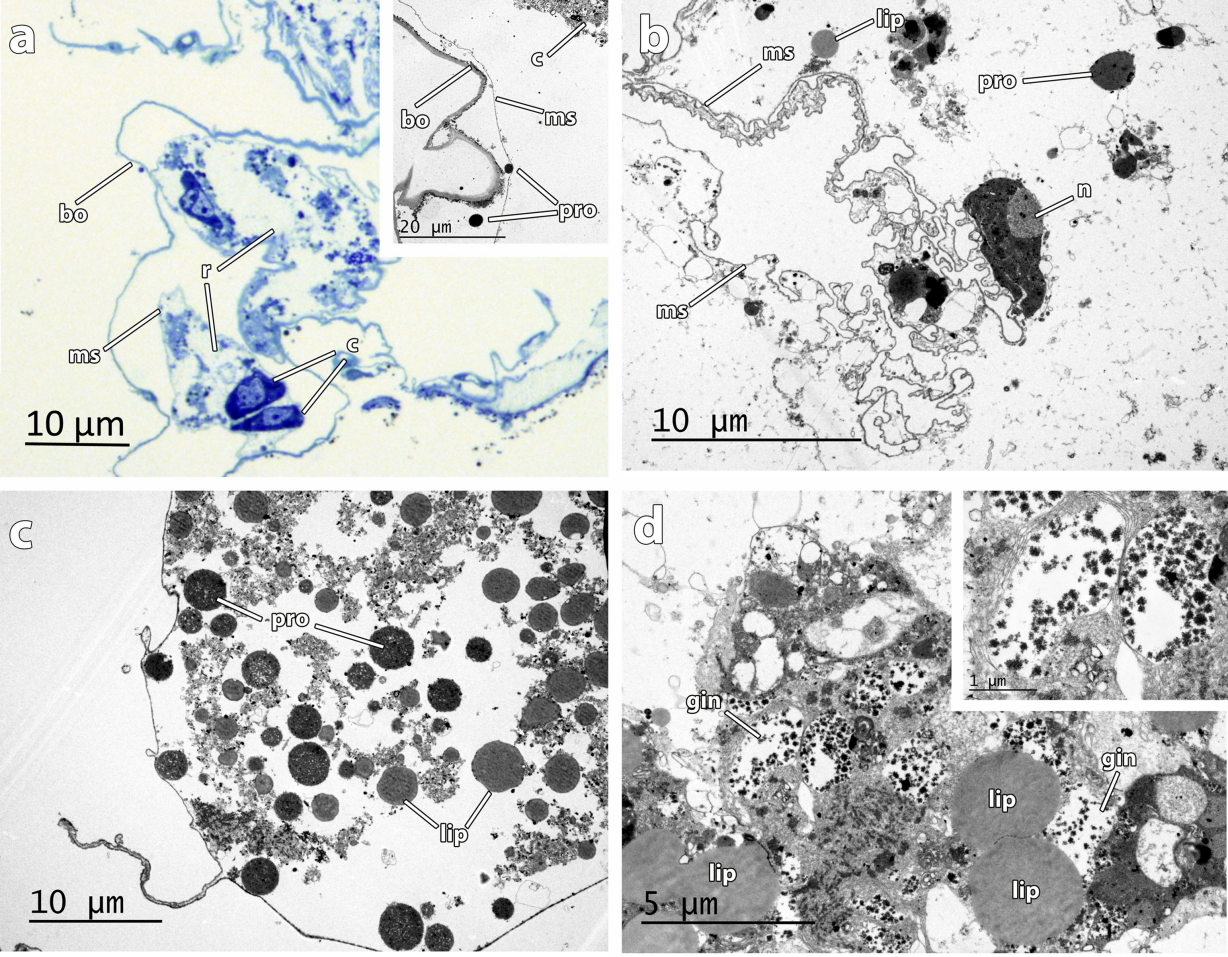
Semithin section and TEM micrographs of *Osedax deceptionensis* transition bone-roots a Eroded bone layer (*bo*) and root mucous sheath (*ms*) containing root cells (*c*). Inset with two protein droplets (*pro*) close to the mucous sheath, **b** Detail of a nucleus (*n*) of a root cell and the highly convoluted root mucous sheath (*ms*). Lipid droplets (*lip*) into the root cell, **c** Vacuolar structure engulfing protein (*pro*) and lipid (*lip*) droplets, **d** Detail of vacuolar structure showing lipid droplets (*lip*) and granular inclusions (*gin*). Inset showing two granular inclusions in detail.

**Fig 4:**
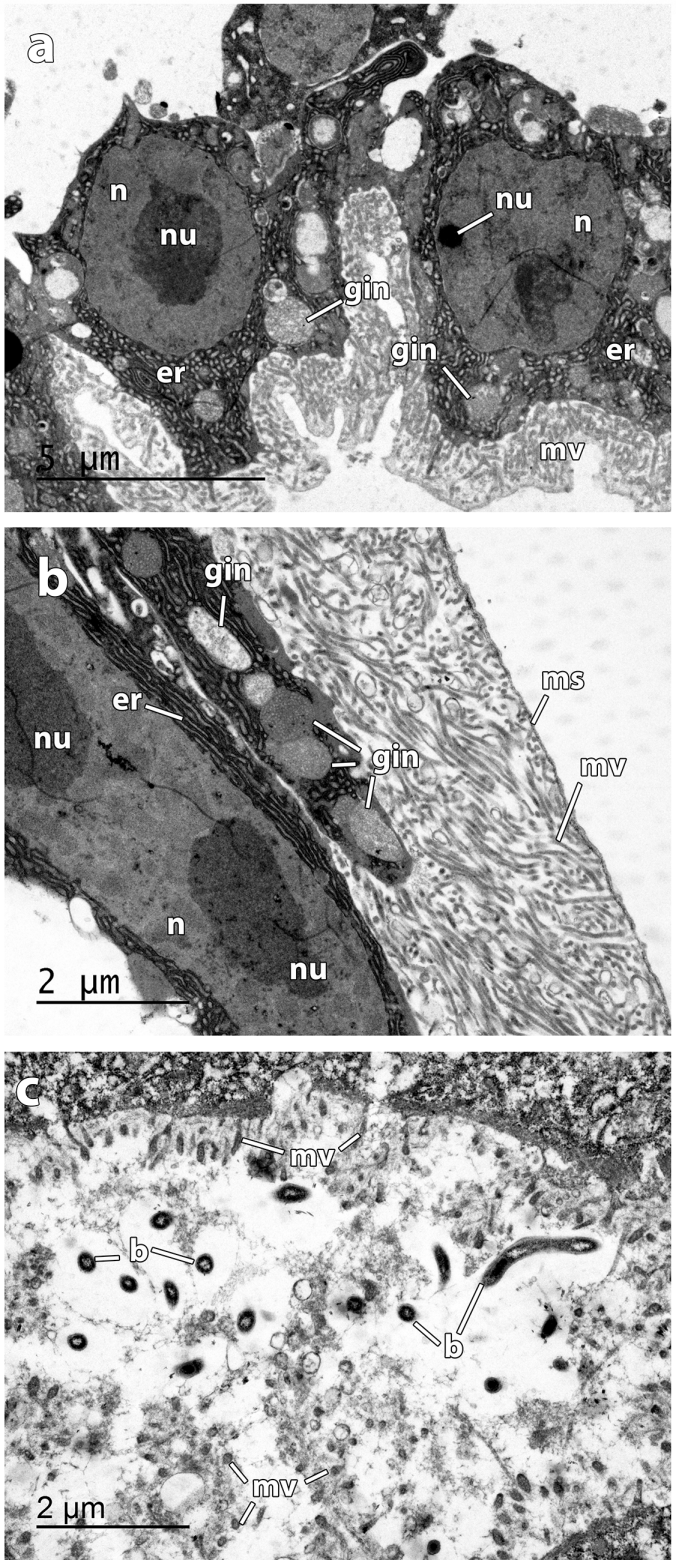
TEM micrographs of *Osedax deceptionensis* epidermis at the roots a Columnar cells of the epidermis at distal roots, showing two nucleus (*n*) with nucleolus (*nu*) surrounded by abundant rough endoplasmic reticulum (*er*) and granular inclusions (*gin*) intermingled. Apical part of cells covered by a dense microvillous layer (*mv*), **b** Detail of a columnar cell, showing the nucleus (*n*) with two nucleolus (*nu*), the rough endoplasmic reticulum (*er*) with granular inclusions (*gin*), and the microvillous layer (*mv*) limited by a mucous sheath (*ms*), **c** Detail of microvillous layer (*mv*) at epidermis close to ovaria with bacteria (*b*) intermingled with microvilli.

**Fig 5:**
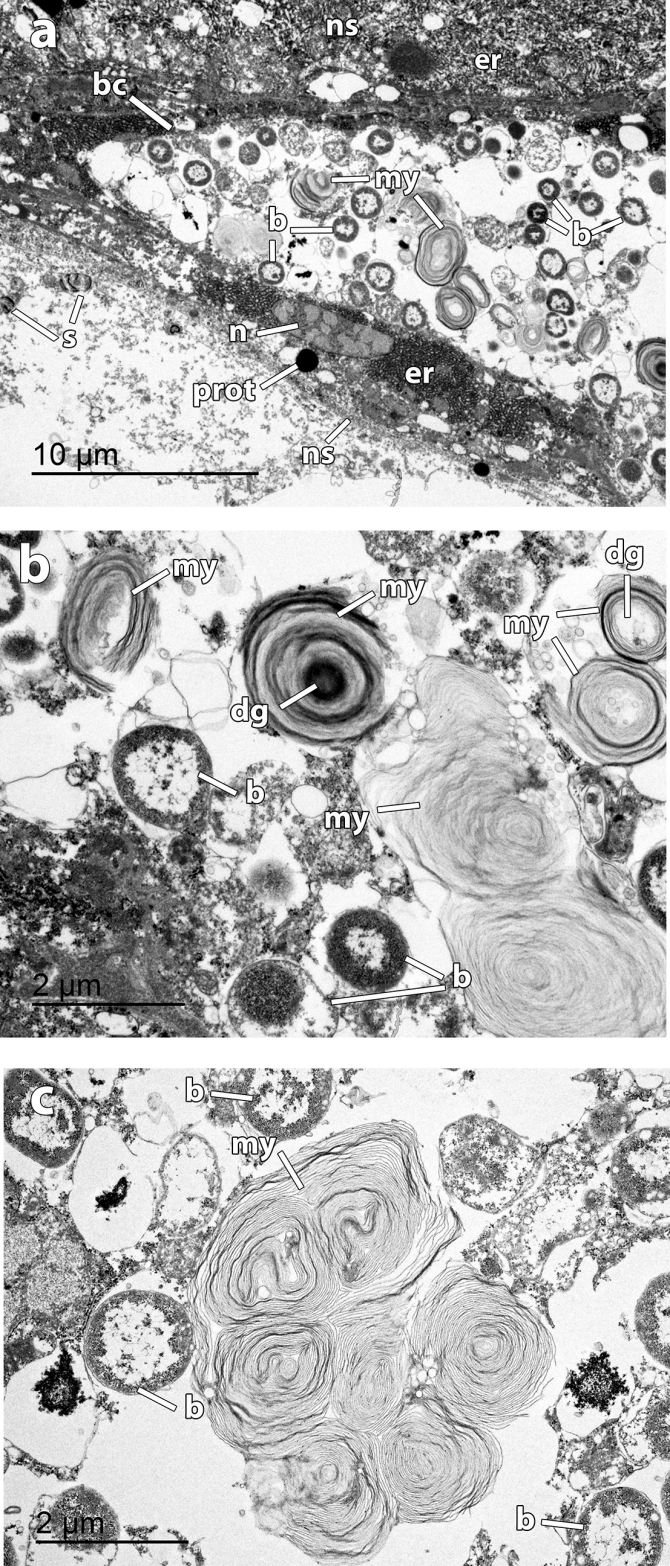
TEM micrographs of *Osedax deceptionensis* bacteriocytes and endosymbionts a Bacteriocyte (*bc*) at roots close to ovaria, surrounded by non-symbiotic cells (*ns*). Non-symbiotic cells (*ns*) with abundant rough endoplasmic reticulum (*er*) and protein droplets (*prot*); notice sperm (*s*) occasionally seen close to bacteriocytes (*bc*). Bacteriocyte (*bc*) containing bacteria (*b*) and myelin bodies (*my*), **b** Detail of bacteria (*b*) inside the bacteriocyte showing different stages of degradation, including earlier stages of degradation (*dg*) and myelin bodies (*my*) surrounding bacteria, **c** Detail of bacteria (*b*) inside the bacteriocyte including a large myelin body (*my*) resulting from the degradation of several bacteria.

**Fig 6:**
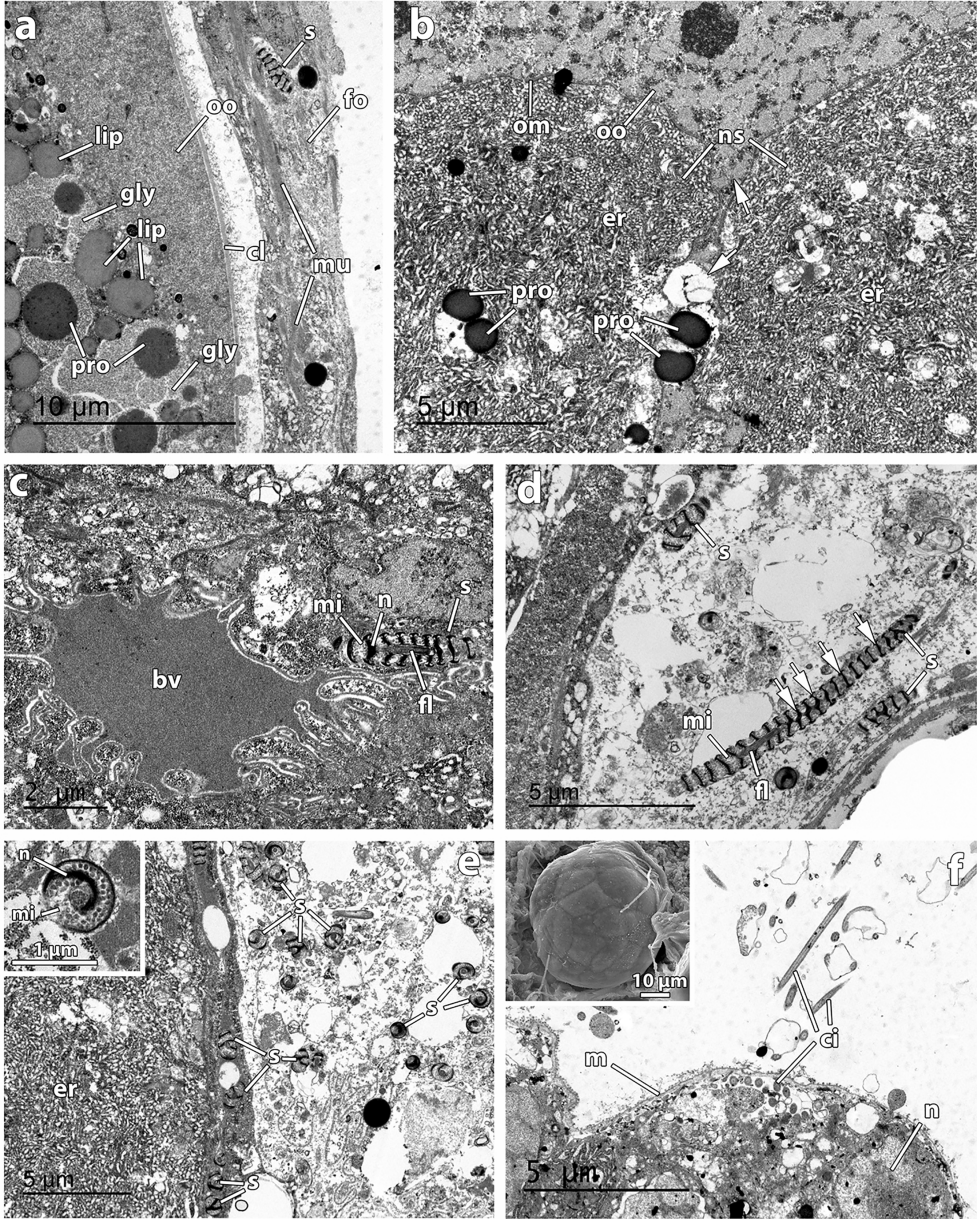
TEM and SEM micrographs of *Osedax deceptionensis* oocytes, sperm, embryos, and larvae a Oocyte (*oo*) in the ovaria packed within a collagenous layer (*cl*) containing yolk with protein (*pro*) and lipid (*lip*) inclusions, and glycogen (*gly*). Oocyte surrounded by a follicle (*fo*) with muscular fibres (*mu*) with sperm (*s*) embedded, **b** Non-symbiotic cells (*ns*) with rough endoplasmic reticulum (*er*) producing proteins (*pro*) transferred to the oocyte (*oo*). Oocyte (*oo*), limited by a membrane (*om*), extending interdigitated cytoplasmic extensions (arrowed), **c** Blood vessel (*bv*) with several expansions close to a sperm (*s*). Notice the helically spiraled nucleus (*n*), the flagellum (*fl*), and mitochondria (*mi*) of the sperm, **d** Sperm (*s*) in longitudinal section. Notice the nucleus (arrowed), mitochondria (*mi*), and flagellum (*fl*), **e** Cluster of sperm (*s*) in transversal section, in an area surrounded by rough endoplasmic reticulum (*er*) close to oocytes. Inset showing a detail of a sperm in transverse section with the nucleus (*n*) and mitochondria (*mi*), **f** Trochophore-like larva, inside the female’s tube, filled with protein and lipid vitellogenic content. Ciliary bands (*ci*) present. Notice a membrane (*m*) limiting the larva and nucleus (*n*) of larval cells. Inset showing a SEM micrograph of a developing embryo inside the female’s tube.

### Material examined

Port Foster, Deception Island (South Shetland Islands), from 6 different minke whale bones –5 of them inside wire cages–experimentally deployed at Whalers Bay, 10 m depth (62°59' 23.7'' S, 60°33' 41.0'' W; Fig 1C, Sta. 1; Table 1). Specimen (CRBA-33236) preserved in 10% formalin and transferred to 70% EtOH: female inside a multilobulated tube. Specimens (CRBA-33237–33239) preserved in glutaraldehyde/osmium tetroxide and mounted for SEM: CRBA-33237, complete female with four palps; CRBA-33238, incomplete female with four palps; and CRBA-33239, tube with embryos inside. Specimens (CRBA-33240–33245), preserved in glutaraldehyde/osmium tetroxide and dissected for TEM: CRBA-33240–33243, tubes with embryos inside (tube of specimen CRBA-33240 belongs to specimen CRBA-33239; tube of specimen CRBA-33241 belongs to specimen CRBA-33245; tube of specimen CRBA-33242 belongs to specimen CRBA-33238); and CRBA-33244–33246, roots and transition between bone-roots. The rest of *O*. *deceptionensis* organisms or pieces of them (n = 18 from Deception Island, n = 2 from South Georgia Island, one specimen vouchered at SIO-BIC A5470) were preserved in 96% EtOH (S3 Table). Material collected by S. Taboada, J. Cristobo, M. Bas, A. Riesgo, J. Moles, C. Angulo, C. Avila (Deception Island) and G.W. Rouse and N.G. Wilson (South Georgia Island).

### Morphological description

Live specimens as four pale white to translucent palps with red margin due to blood vessels (Fig 2A). Four free (not fused at their base) palps, smooth and without pinnules even at high magnification (not shown), of equal length ca. 1.40 mm, 100 μm wide. No oviduct observed. Hemispherical (in small females) to elongate and multilobate (in larger females) translucent gelatinous tubes up to 13 mm (Fig 2B–2D). Several tubes containing embryos/larvae in their lumen (83–123 μm long to 73–94 μm wide; n = 14), which appeared to be either concentrated at the base and/or at the most distal parts of the tube (Fig 2C–2E). No dwarf males identified inside the tubes. Trunk smooth, 1.42 mm long, 425 μm wide in preserved specimens, whitish in live specimens, white opaque after preservation. Mouth and gut absent. Ovisac and very lobate roots, greenish in life, retaining color after preservation. Several spherical eggs not measured.

### Ultrastructural description

Four general regions (the crown, the trunk, the ovisac and the roots) comprised the body of *O*. *deceptionensis*. The roots of *O*. *deceptionensis* appeared as convoluted lobes in direct contact with the bone (Fig 3) and have an epidermis with columnar cells (Fig 4) surrounded by a mucous sheath (Fig 3). The trophosome (comprised by non-symbiotic cells and bacteriocytes; Fig 5) and ovary (Fig 6) were contained in the ovisac, without any septa separating it from the roots. Here we describe the ultrastructure of significant features of the roots, trophosome, and ovary of *O*. *deceptionensis*, including sperm occurring in the tissue next to the ovisac.

In the transition between the bone and the roots, eroded bone appeared in intimate relation with a highly convoluted mucous sheath in the roots containing amoeboid cells ca. 5 μm in maximum diameter (Fig 3A, 3a inset). Protein and lipid droplets, presumably obtained after bone degradation by the worm, appeared to be released to the interior of the animal roots (Fig 3A inset, b) and then engulfed and packed into vacuolar structures by root cells (Fig 3C and 3D). Apart from protein inclusions and lipid droplets, granular inclusions of unknown origin also appeared packed into the vacuolar structures within cells in the periphery of the roots (Fig 3D, 3d inset).

The epidermis of *O*. *deceptionensis* at distal roots was composed by large columnar cells ca. 10 μm in maximum length, with their apical part covered by a dense microvillous layer (Fig 4A and 4B); these microvilli appeared to be limited by a mucous sheath (Fig 4B). Epidermal cells contained a large, elongated and binucleolated nucleus surrounded by abundant rough endoplasmic reticulum, and granular inclusions intermingled (Fig 4A and 4B). In the ovisac region, the disposition of columnar cells and microvilli was similar to that in the distal roots, but here occasional rod-shaped bacteria appeared in close relation to microvilli (Fig 4C).

No bacteriocytes could be observed in the distal part of the roots of any specimen. Instead, bacteriocytes observed in the trophosome close to the ovisac appeared mixed with non-symbiotic cells. Bacteriocytes were large ovoid cells (Fig 5A) containing abundant roundish symbionts, commonly found within a vesicle; symbionts ranged from intact bacteria to completely degraded bacteria with myelin bodies surrounding them (Fig 5A–5C).

Follicles containing large muscle bundles and cells with large numbers of vesicles and with sperm embedded surrounded the oocytes in the ovarian tissue (Fig 6A). Follicle cells contained sperm embedded (Fig 6A). Oocytes, enveloped by a collagenous layer, occurred in the ovary and contained yolk apparently of protein and lipid nature and also glycogen (Fig 6A). Non-symbiotic cells, containing massive rough endoplasmic reticulum, surrounded the oocytes and were producing vacuoles seemingly captured by the oocyte interdigitated cytoplasmic expansions (Fig 6B). Blood vessels with several expansions went through the ovarian tissue (Fig 6C). The sperm of *O*. *deceptionensis* found in the ovisac were flagellated with elongate heads with an electron-dense helically spiraled nucleus alternating with multiple mitochondria (Fig 6C–6E, 6 inset). Embedded sperm within cells were primarily found close to oocytes sometimes in numerous clusters (Fig 6E) although they were also present even at the most distal parts of the female’s roots (not shown).

Embryos and larvae at different developmental stages were present in the lumen of female tubes: from developing elliptical embryos (Fig 6F inset) to trochophore-like larvae, with cilia (Figs 2E and 6F). Larvae were filled with protein and lipid yolk, with no trace of bacterial symbionts. A single female tube contained up to 81 embryos/larvae.

### Phylogenetic analyses

**Fig 7:**
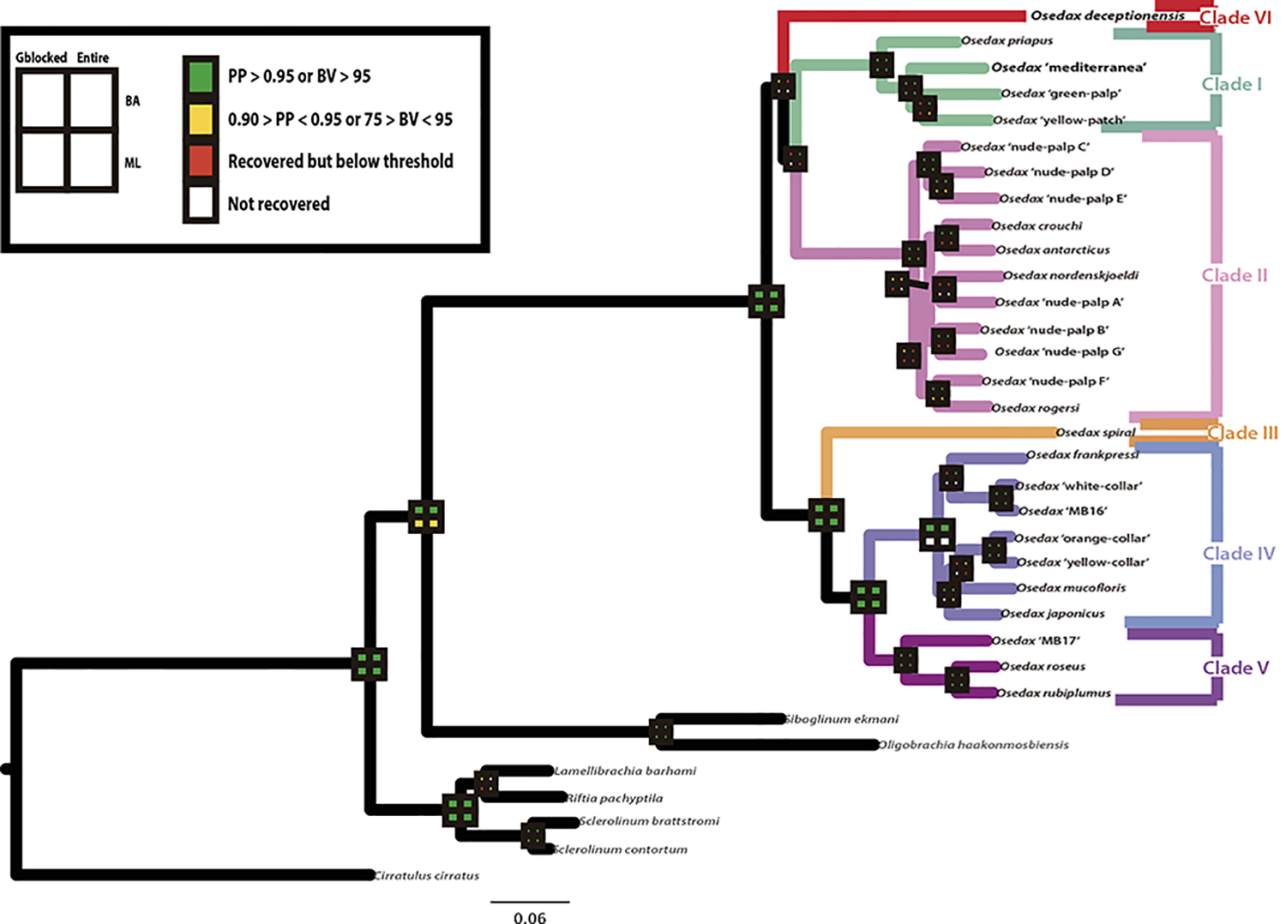
Phylogenetic tree of *Osedax* based on the concatenated analyses (Gblocked dataset) of *COI*, *16S*, *18S*, *28S*, and *H3* from Bayesian inference analysis (BI). Squares on nodes refer to support retrieved from 4 different analyses: upper, left gblocked alignment under BI; upper right, complete alignment under BI; bottom left, gblocked alignment under ML; bottom right, complete alignment under ML. Green squares indicate posterior probability values (PP) > 0.95; bootstrap support (BS) > 95, yellow squares indicate 0.90 > PP < 0.95; 75 > BS < 95), red squares indicate clade recovered but below thresholds) and white indicates clade not recovered. *Osedax deceptionensis* and *O*. ‘mediterranea’ are indicated in bold. The six major *Osedax* clades previously defined by [11] and [12] are highlighted.

The consensus tree obtained from the Bayesian (BI) analysis on the Gblocked concatenated alignment, which also summarizes the support recovered after conducting BI and ML analyses on the Gblocked and the entire dataset alignments, is shown in Fig 7. The Gblocked concatenated alignment consisted in 4,415 characters, 1,596 of *18S*, 1,004 of *COI*, 963 of *28S*, 481 of the *16S*, and 371 characters of *H3*, while the entire dataset consisted of 4,695 characters, 1,689 of *18S*, 1004 of *COI*, 1,124 of *28S*, 507 of the *16S*, and 371 characters of *H3*. Both the BI and the ML analyses recovered a tree in which a monophyletic *Osedax* was robustly supported, with internal structure mostly resolved (Fig 7). The BI and ML analyses inferred similar topologies and most disagreements involved poorly supported clades. The major clades (lineages) previously defined within *Osedax* [11, 12] were recovered with high support. Despite of the addition of two additional genes and the use of a larger fragment of *COI* for *O*. *deceptionensis* in the present analyses, the relationship between the major clades remained mostly unresolved, except for the already recovered close relationship between Clades III, IV and V. The BI and ML analyses of the entire dataset recovered the sister group relationship between Clades I and II, which received high support in the BI analysis. Conversely, ML analysis of the Gblocked matrix recovered a closer relationship of Clade I with Clades III, IV and V, albeit with low support. None of the analyses provided support for the actual position of *O*. *deceptionensis*, which was recovered as sister to the remaining clades in the ML analyses but as sister to Clades I and II in the BI analyses. These results suggest an isolated position of *O*. *deceptionensis* within the extant *Osedax* diversity (Clade VI; Fig 7). All analyses agreed in supporting the inclusion of the newly found *O*. ‘mediterranea’ within Clade I, closely related to the *O*. ‘green palp’ and *O*. ‘yellow patch’. The three OTUs are in turn supported as the sister taxa to *O*. *priapus* (Fig 7). No major incongruence was found between trees obtained using genes treated separately and the results after the concatenated analyses.

Genetic distance between *O*. *deceptionensis* and the rest of the *Osedax* taxa for the *COI* ranged from 15.8 to 24.0% and from 20.1 to 35.7% for the *p*-distance and the K2p, respectively (S4 and S5 Tables). Distances between *O*. ‘mediterranea’ and the rest of the *Osedax* species ranged from 14.7 to 22.0% and 21.1 to 31.7% for the *p*-distance and the K2p, respectively (S4 and S5 Tables).

**Fig 8:**
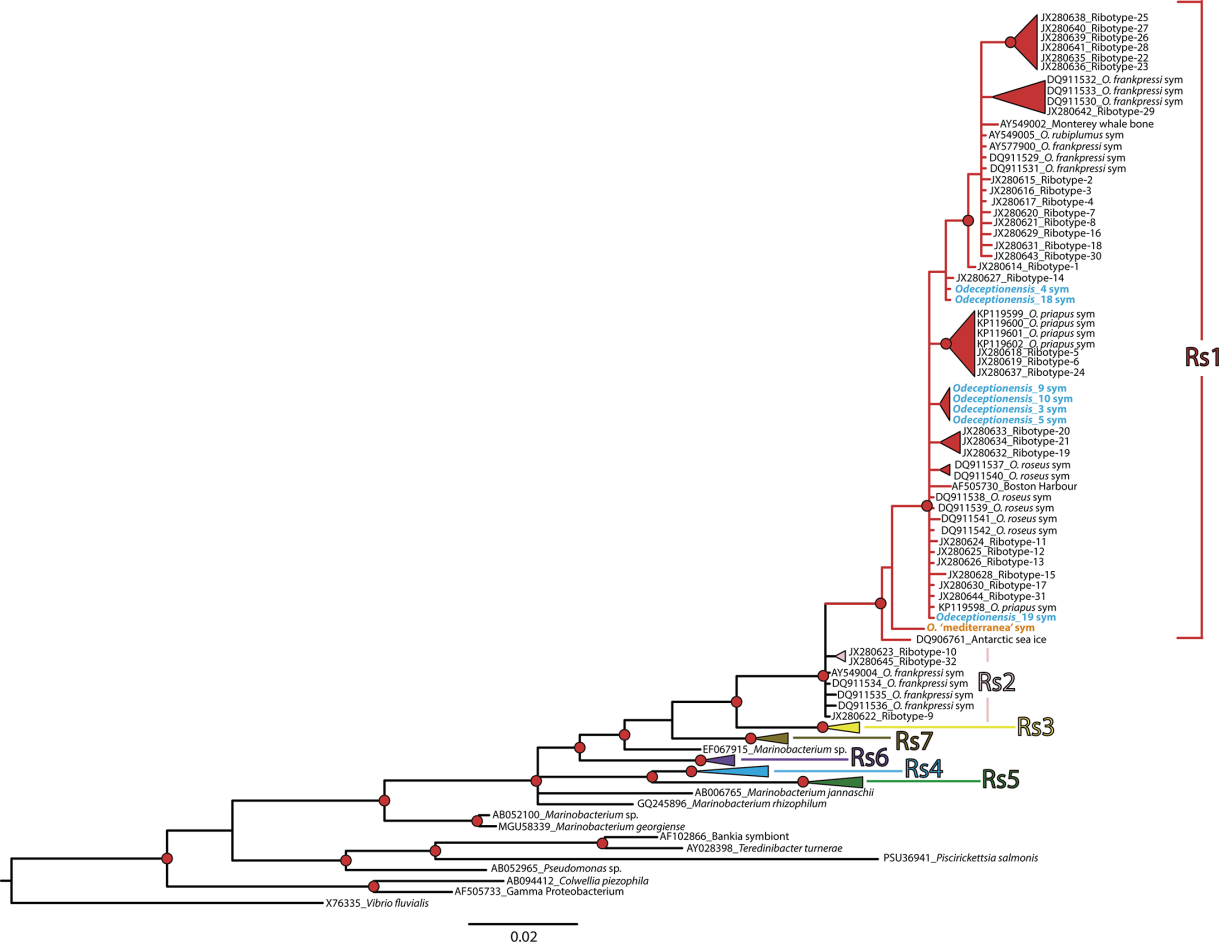
Phylogenetic tree of *Osedax* endosymbionts resulting from the *16S* marker, using Bayesian inference (BI). Nodes with posterior probability values > 0.95 marked by red dots. Endosymbionts of *Osedax deceptionensis* and *O*. ‘mediterranea’ are indicated in bold in blue and orange, respectively. Seven major *Osedax* endosymbiont clades or ribospecies (Rs1–Rs7) are distinguished following [32].

The *16S* dataset including the newly-sequenced *Osedax* endosymbionts was 680 bp. The BI analysis recovered clades congruent with those reported by [32]. Out of the seven individuals of *O*. *deceptionensis* sequenced, we found four different ribotypes, all of them belonging to the ribospecies Rs1 (Fig 8). The Oceanospirillales ribospecies found in *O*. ‘mediterranea’ also belonged to ribospecies Rs1 (Fig 8).

### Divergence time estimation

**Fig 9:**
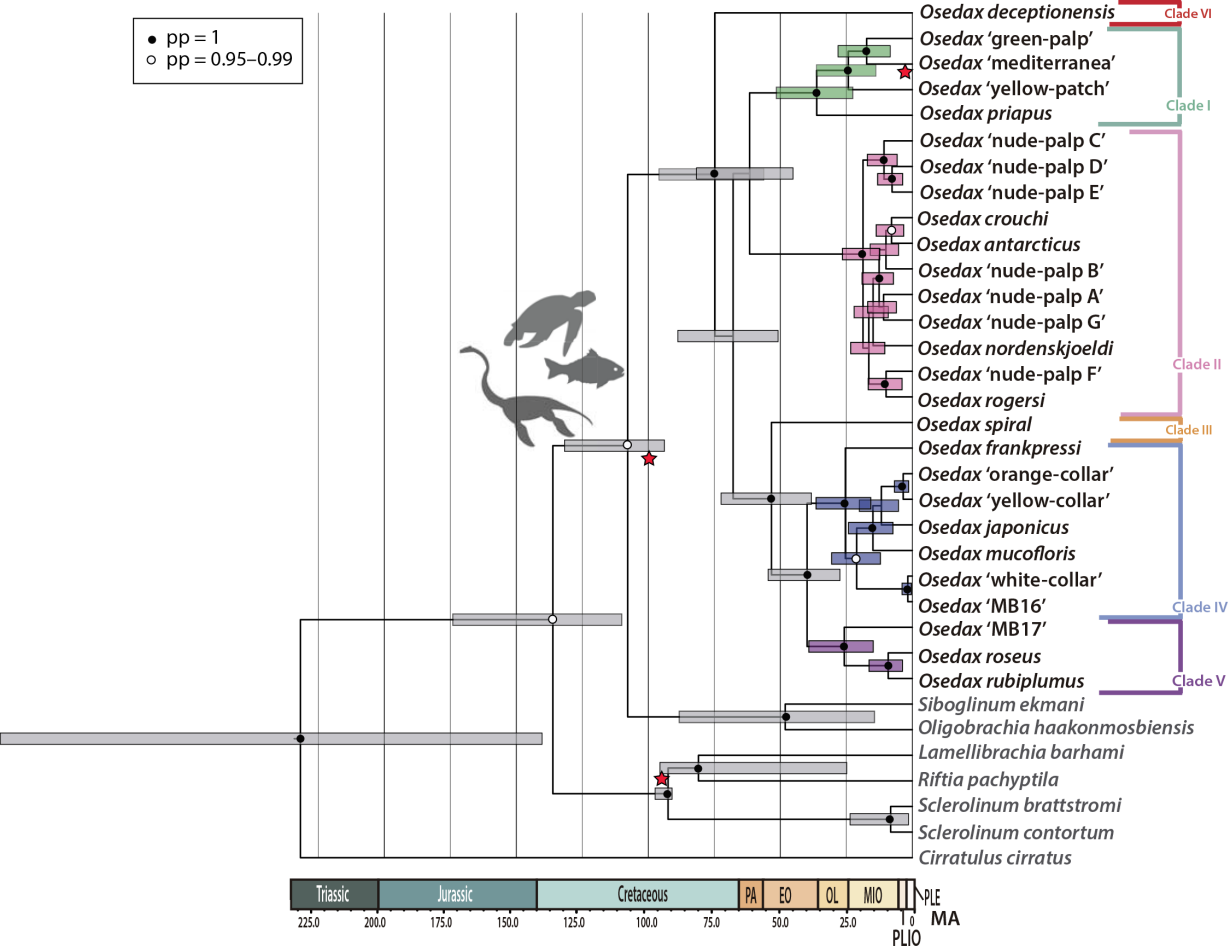
*Osedax* time-calibrated phylogeny based on the Gblocked concatenated (*COI*, *16S*, *18S*, *28S*, and *H3*) BI analysis. Fossil calibration points indicated by red stars (see Material and Methods section for details). Bars at nodes indicate the 95% highest posterior density (HPD) interval. Nodes marked by black dots indicate posterior probability values of 1, while white dots indicate posterior probability values of 0.95–0.99.

According to our results, the ancestor of *Osedax* split from its siboglinid sister group during the Middle Cretaceous, ca. 108 My (132–94 Ma), while the most recent common ancestor of the extant *Osedax* species appeared during the Late Cretaceous, ca. 74.8 Ma (96–56 Ma; Fig 9). Clade II, which includes the deep-water Antarctic species, appears to have undergone a rapid diversification in the Early Miocene, around 20 Ma (26–12 Ma), while the last common ancestor of *O*. ‘mediterranea’ appeared ca. 17.3 Ma (28–8 Ma).

### Population genetic analysis

**Fig 10:**
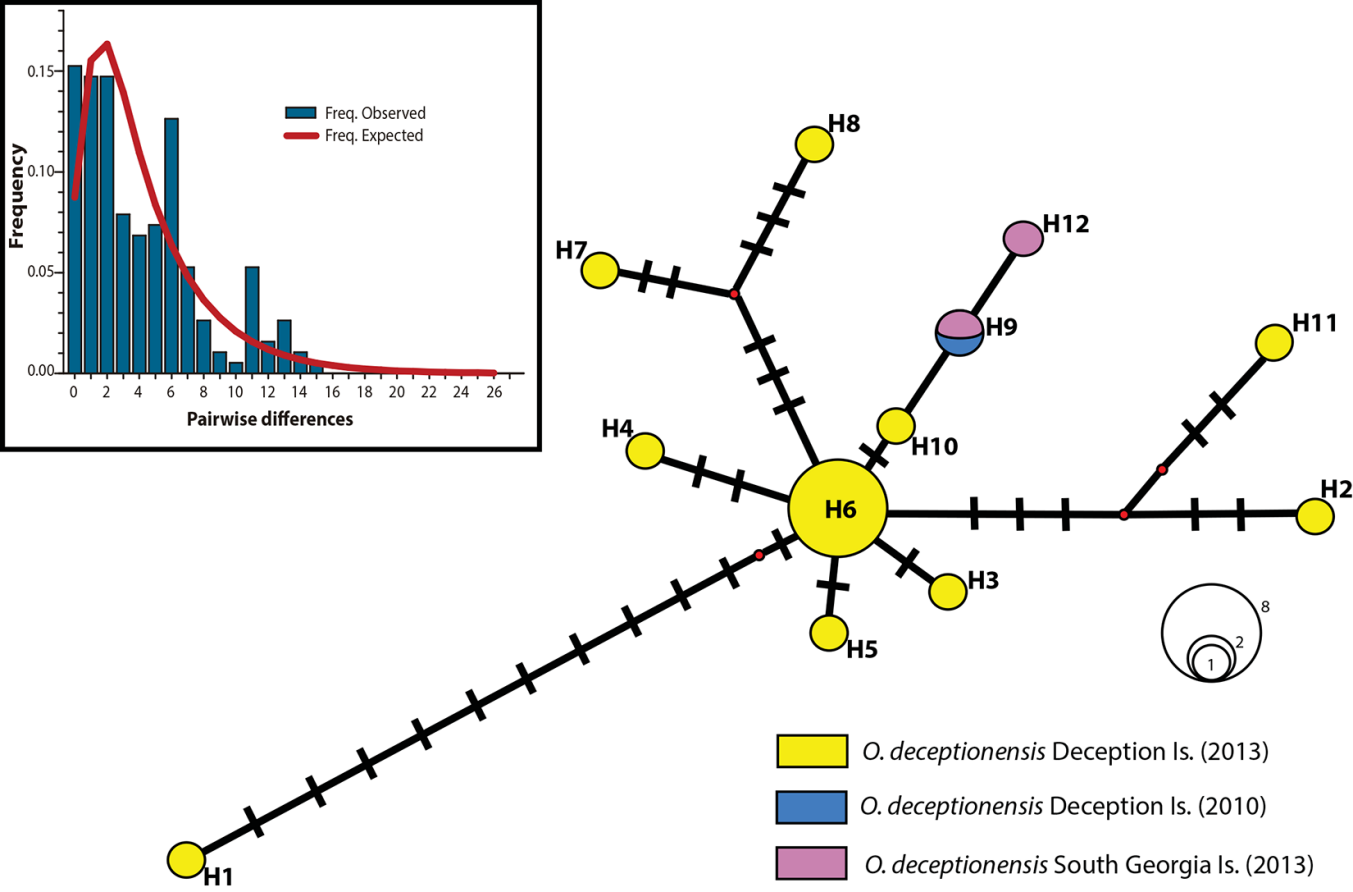
Haplotype network for *Osedax deceptionensis* from 20 individuals, including 18 individuals from Deception Island and 2 individuals from the vicinity of South Georgia Island. In yellow, haplotypes from Deception Island collected in 2013; in blue, the haplotype from Deception Island collected in 2010 [12]; in purple, haplotypes from South Georgia Island collected in 2013; in red, missing inferred haplotypes. Inset, observed mismatch distribution (vertical bars) and expected frequency distribution (line), assuming population size changes.

A total of 445 bp (379 bp excluding missing data) of *COI* were obtained from 20 individuals of *O*. *deceptionensis* collected in Deception Island (n = 18) and South Georgia Island (n = 2). In total, 28 variable sites (6%) and 12 different haplotypes were found in the dataset. Nine haplotypes were private from Deception Island (H1–H5, H7–H8, H10–11), one from South Georgia Island (H12) and two haplotypes were shared between the two localities (H9, present in one individual from each locality) and H6 was present in 8 individuals in Deception Island (40% of the total sample) (Fig 10). Haplotype diversity value was 0.8474 and the haplotype mismatch distribution followed a unimodal pattern (Fig 10 inset), with a Tajima’s *D* value of -1,9292 (P < 0.05). The genetic divergence within *O*. *deceptionensis* ranged from 0.0 to 3.9% and from 0.0 to 4.2% for *p-*distance and K2p models, respectively (average of 1.11% of genetic divergence for both distances; S6 and S7 Tables).

## Discussion

### Biogeographic considerations

Our study confirms a broader range for *Osedax deceptionensis* in the shallow-waters of the Southern Ocean, by extending its current distribution to the Subantarctic waters of the South Georgia area. This species occurred in our study at moderate abundances in some of the bones experimentally deployed at Port Foster, Deception Island’s bay, even on bones not protected against potential predators by wire cages. Port Foster is an enclosed drowned caldera with a maximum depth of 180 m, connected to the open sea by a narrow, shallow opening with very limited water exchange [44]. The fact that the *O*. *deceptionensis* population at Deception Island appears to be genetically connected to the Subantarctic South Georgia region, an area ca. 1,600 km north east, may suggest that these organisms are good dispersers, probably thanks to their lecithotrophic larvae, as already proposed in previous studies (*e*.*g*., [13]). Tajima’s D negative value and the unimodal mismatch distribution observed for *O*. *deceptionensis* suggest a recent population expansion [45, 46]. Similar results have also been reported for *O*. *rubiplumus* and *O*. *rogersi*, while the analyzed populations of *O*. *antarcticus* and *O*. *crouchi* have been postulated to be at demographic equilibrium [10, 13].

In the Southern Ocean and Subantarctic waters, appropriate habitat for *O*. *deceptionensis* might be common since there is a long list of marine mammals and birds living in this region. Apart from whale bones–investigated in the present study as well as in most of the studies conducted so far–, the presence of colonies of penguins and pinnipeds, among other vertebrates, could represent a source of bones suitable as a substrate for *O*. *deceptionensis* settlement [22, 47, 48]. Therefore, we hypothesize that *O*. *deceptionensis* has a widespread distribution all along the shallow-water Antarctic and Subantarctic shores, which should be corroborated in future studies.

The relatively frequent occurrence of *O*. *deceptionensis* at very shallow waters in the Southern Ocean contrasts with the difficulties to collect congeneric organisms at similar depths in the Mediterranean. Our findings confirm the occurrence of *Osedax* (*O*. ‘mediterranea’) for the first time in the Mediterranean Sea, although the presence of *Osedax* in the Mediterranean had been already advanced by [41] based on borings presumably made by *Osedax* in whale fossils from Italy, laid on a muddy seafloor at 90 m depth ca. 3 Ma ago. In the Mediterranean, [49] conducted similar experiments to those here reported, close to the area where *O*. ‘mediterranea’ was collected, without finding any evidence of *Osedax* colonization in any of the bones of different mammals (minke whale, pig, and cow) monitored over a year. The quick decomposition of the lipid content in the bones in warmer temperatures could be behind the absence of *Osedax* in this geographic area at depths shallower than 50 m. In the shallow-waters where *Osedax* are commonly found, temperature is usually low (-1–15°C: [12, 50–54]. Such relatively low temperatures are clearly out of the range of the seawater temperature (11.8–22.2°C) recorded in our Mediterranean experiments including also those in [49]. Therefore, the serendipitous finding of *O*. ‘mediterranea’ colonizing just one of the several bones we deployed at ca. 50 m depth might be due to the important influence that these bones received from deeper, colder waters from the Blanes submarine canyon [55], an area where more abundant populations of *Osedax* would presumably occur in suitable bone substrates.

### *Osedax* Phylogeny: hosts and symbionts

Recent studies have investigated the phylogenetic relationships in *Osedax*, finding little or no support for many relationships within the genus, including the position of *O*. *deceptionensis*, suggesting that further molecular data was required to fully resolve *Osedax* phylogeny [4, 10, 12]. Unfortunately, the addition of two more nuclear markers and the inclusion of a larger *COI* fragment for *O*. *deceptionensis* in the present study had little effect on resolving its phylogenetic relationships. However, our results suggest that *O*. *deceptionensis* may be a unique lineage within *Osedax* with no clear affinities among the major clades in the genus. Additionally, our analyses consistently recovered, with high support, the Mediterranean *Osedax* as a member of Clade I and confirm that shallow-water species of relatively close areas (the North Atlantic and the Mediterranean), namely *O*. *mucofloris* and *O*. ‘mediterranea’, originated independently. *Osedax* ‘mediterranea’ belongs to the same clade as *O*. *priapus*, the only *Osedax* without dwarf males reported so far [4], but also with *O*. ‘yellow patch’, which shows the typical sexual dimorphism reported in the genus (see [4]). Finding of the males of the Mediterranean species may help to elucidate the evolution and polarity of dwarf males within the clade.

Interestingly, neither our analyses nor those of [4] recovered the ‘Antarctic clade’ (*O*. *antarcticus*, *O*. *crouchi*, *O*. *rogersi*, and *O*. *nordenskjoeldi*) within Clade II as recently proposed by [10], which may suggest a more complex scenario on the origins of deep-water Antarctic *Osedax*.

All endosymbiont ribotypes found in *O*. *deceptionensis* and *O*. ‘mediterranea’ fell within ribospecies Rs1, a clade that was so far only characterized by deep-water endosymbionts [4, 32]. Thus, Oceanospirillales endosymbionts occurring in shallow-water *Osedax* comprise now a polyphyletic assemblage of ribospecies including Rs1, Rs3, and Rs7 [32], which suggests that there is no segregation between shallow- and deep-water Oceanospirillales ribospecies in *Osedax*.

### A timeframe for the diversification of *Osedax*

Our time estimates suggest that *Osedax* diverged from other siboglinids in the Middle Cretaceous (ca. 108 Ma), a period in which bones of large reptiles, including plesiosaurs, sea turtles and probably teleost fishes would have provided habitat and sustenance for *Osedax* [36]. Our estimates further show that the most recent common ancestor of extant *Osedax* appeared during the Late Cretaceous (ca. 74.8 Ma), indicating that the radiation of *Osedax* would be concomitant with the occurrence of large marine reptiles and teleost fishes in the oceans [56, 57] rather than with the origin and radiation of large archaeocete cetaceans during the Eocene (ca. 50 Ma; [58]). The upper end of the posterior interval (56 Ma), however, does overlap with the older evidence of archaeocete cetaceans. Therefore, a close link between the extant diversity of *Osedax* and the origin of whales, as proposed by [11] and [36], cannot be ruled out.

A rapid diversification of Clade II, including recently described species from Antarctica, took place during the Early Miocene, about 20 Ma. Splits between the Antarctic species and their sister groups were dated between the middle Miocene and the middle Pliocene. The intensification of the Antarctic Circumpolar Current that occurred approximately 14 Ma [59], may have contributed to the isolation of the Antarctic populations and eventually led to the formation of the local species. As for *O*. ‘mediterranea’, our estimations suggest that its last common ancestor lived ca. 17.3 Ma. The closing of the Gibraltar strait dated at 5.96 Ma. and the subsequent desiccation of the Mediterranean Basin [60], drove the extinction of marine life from most of the basin. Therefore, we hypothesize that the ancestors of *O*. ‘mediterranea’ originated in the Atlantic and subsequently colonized Mediterranean waters, following the reopening of the Strait of Gibraltar, dated at 5.3 Ma, which restored the water exchange between the Atlantic and the Mediterranean [61].

Divergence time estimation is based on a series of assumptions and hence a certain caution should always be exerted on the interpretation of the results. The present estimates for the diversification of *Osedax*, however, are based on the largest taxonomic sample gathered to date for this group and, for the first time, they incorporate fossil calibration points, at the same time that integrate the available information on substitution rates used in former analyses [1, 11]. New relevant fossil evidence found in the near future and a more complete view on the current *Osedax* diversity shall corroborate or reject the evolutionary scenario proposed in the present study.

### New morphological insights into *O*. *deceptionensis*

*Osedax deceptionensis* was originally described using a single mature female collected from one whale bone at Deception Island [12]. Due to the limited information gathered from this unique individual, there was a need to complement the morphological description of this species. *In vivo* observations of several organisms allowed us to confirm that palps of *O*. *deceptionensis* were translucent but had thin red lines (= blood vessels) running along their margins, similar to those reported for the also Antarctic *O*. *rogersi*, *O*. *antarcticus*, and *O*. *crouchi*, although in the two latter palps are striped [10, 12]. Interestingly, palps in *O*. *deceptionensis* are not fused as opposed to the rest of Antarctic *Osedax*, which have at least ca. 50% of their palps fused [10, 12]. As a common trait, *O*. *deceptionensis* and the rest of Antarctic *Osedax* share smooth palps under the light microscope but differ in that no rugose appearance at higher magnification was observed for *O*. *deceptionensis*.

After our measurements on newly preserved material of *O*. *deceptionensis*, we revised the estimate for the maximum length (palps + trunk) of these organisms from 1.2 mm [12] to 2.82 mm. Thus, after the recent discovery of *O*. *priapus*, *O*. *deceptionensis* would now be the second smallest *Osedax* described so far (see Table 1 in [4] and [10] for comparison). As for the tube, its shape appeared to vary depending on the size of the individuals: the hemispherical tube recorded for the type species [12] was also observed in small-sized individuals in this study (Fig 2B), while elongated and even multilobate larger tubes were recorded in larger females (Fig 2C and 2D). To our knowledge, multilobate tubes are reported here for the first time for any *Osedax* (see [10]). Interestingly, embryos and larvae were present only in the lumen of large multilobate tubes, although not homogeneously distributed (Fig 2C and 2D). A previous study on *O*. ‘orange-collar’ confirmed that fertilized oocytes are released to the water column through the oviduct and that early trochophores develop after two days under laboratory conditions [7]. Thus, although from our observations it could be inferred that *O*. *deceptionensis* may brood their larvae as suggested for *O*. *japonicus* [14, 53], this is likely to be a laboratory artifact since after recovery of bones they were kept in their tanks for more than two weeks.

Several aspects related to the bone bioerosion, endosymbiosis with bacteria, and the reproductive system have been characterized at cellular level in *Osedax* [1, 3, 5, 6, 8, 15, 53, 62, 63]. In order to increase the knowledge on these aspects in a species belonging to a clade never investigated before, which in turn is present in a totally different environment (Antarctic and Subantarctic shallow-waters), we analyzed *O*. *deceptionensis* at the ultrastructural level. Our observations indicated that roots are most likely involved in nutrient uptake, as already proposed by [62]. In some of the transition areas between the bone and the roots, no epidermis was observed in direct contact with bone. Instead, a highly convoluted mucous sheath, similar to that reported in other congeneric OTUs [1, 8, 53, 64], appeared in intimate contact with the bioeroded bone. The protein and lipid droplets, presumably mobilized after the degradation and dissolution of the bone [8], accumulated in *O*. *deceptionensis* in vacuolar structures (Fig 3C), which is reported here for the first time in *Osedax*. The fate of these nutrient accumulations, that will be used by endosymbionts to proliferate and/or will be used directly by the host without intermediate mediation of bacteria [8, 63], remains however unsolved.

As in *O*. ‘green palp’, the epidermis of the root of *O*. *deceptionensis* was covered by a dense layer of microvilli and the elongated nucleus of columnar epidermal cells was massively surrounded by rough endoplasmic reticulum with granular inclusions intermingled [62]. Rod-shaped bacteria were observed in association with the microvilli of the epithelium close to the ovisac in *O*. *deceptionensis*, also observed in *Osedax* ‘MB3’ and *O*. *mucofloris* [6, 53] but not in *O*. ‘green palp’ [62].

In our study, bacteriocytes occurring in the roots close to the ovisac in *O*. *deceptionensis* appeared to be very similar to those described for *O*. ‘green palp’ in a comparable region of the roots. In the bacteriocytes of *O*. *deceptionensis*, roundish bacteria and myelin figures were found concomitantly in bacteriocytes of the ovisac, whereas in *O*. ‘green palp’ bacteriocytes at the roots contained intact sometimes dividing rod-shaped bacteria, and ovisac bacteriocytes contained mostly degraded bacteria in myelin figures [63].

The general features of oocytes and sperm in *O*. *deceptionensis* are similar to those recently reported for *O*. *rubiplumus*, *O*. *frankpressi*, *O*. ‘green palp’, and *O*. ‘yellow collar’ [15]. Here, we document the presence of vitellogenic oocytes surrounded by a follicle in the ovarian tissues of *O*. *deceptionensis*, as already noted for *O*. ‘green palp’ [15]. This follicle was muscularized and occasionally showed embedded sperm (Fig 6A). The massive rough endoplasmic reticulum of the non-symbiotic cells surrounding oocytes seems to be behind the production of the protein inclusions later incorporated by oocytes (Fig 6B). Although the presence of males could not be documented, the sperm ultrastructure in *O*. *deceptionensis* was assessed in the ovarian tissue of females. Sperm of *O*. *deceptionensis* were largely similar to those described previously [15] as was the presence of sperm in different orientations (Fig 6E) in the vicinities of oocytes.

### Further directions

The present study provides novel information on *O*. *deceptionensis*, which prove to be a novel clade within the genus. We also report for the first time the presence of extant species of the genus in the Mediterranean. Knowledge about *O*. *deceptionensis* is further complemented with additional biogeographic, phylogenetic, morphological and ultrastructural information. Future studies should be directed to corroborate the occurrence of *O*. *deceptionensis* across the shallow-water Southern Ocean and Subantarctic and to establish whether *O*. *deceptionensis* also occurs in South America. Hopefully, the present study will spur the description of the presence and the morphological characters of males in the tube of females of *O*. *deceptionensis*, since not a single male could be identified even after examining several of the tubes collected at Deception Island. As for *O*. ‘mediterranea’, most questions still remain unsolved about its morphological and ultrastructural characters as well as its geographic and bathymetric distribution. Subsequent studies should be directed to establish whether this species only occurs in the shallow Western Mediterranean and/or it is also found at deeper waters in other basins (*e*.*g*. Eastern Mediterranean, Atlantic Ocean).

## Supporting Information

S1 Table PCR temperature profiles and mixtures. (DOCX)

S2 Table Taxa included in the molecular phylogenetic analyses (*Osedax* and *Osedax* endosymbionts) with NCBI GenBank accession numbers. Bold indicates new sequences. ^a^Sequence obtained from *O*. *deceptionensis*_4; ^b^Sequence obtained from *O*. *deceptionensis*_19. ^c^Sequences obtained from *O*. *deceptionensis*_3–5, *O*. *deceptionensis*_9–10, and *O*. *deceptionensis*_18–19. See S3 Table for further details on specimen assignation.
(DOCX)

S3 Table Information about the *COI* sequences used in the haplotype network, including the code of the individual, area and year of collection, haplotype assigned, NCBI GenBank accession number, and the part of the organism used for the DNA extraction. ^a^See Table 1 for further details. ^b^See Fig 8 (haplotype network) for further details.
(DOCX)

S4 Table *COI* divergence values (*p-*distance) between *Osedax* species and OTUs. (DOCX)

S5 Table *COI* divergence values (Kimura 2 parameters) between *Osedax* species and OTUs. (DOCX)

S6 Table *COI* divergence values (*p-*distance) between individuals of *Osedax deceptionensis*. *See S3 Table for further details.
(DOCX)

S7 Table *COI* divergence values (Kimura 2 parameters) between individuals of *Osedax deceptionensis*. *See S3 Table for further details.
(DOCX)
