# Supplementary material for: Bone-Eating Worms Spread: Insights into Shallow-Water Osedax (Annelida, Siboglinidae) from Antarctic, Subantarctic, and Mediterranean Waters
Source: PLoS One. 2015 Nov 18;10(11):e0140341. doi: 10.1371/journal.pone.0140341 (PMC4651350; doi:10.1371/journal.pone.0140341)
Supplement: S1 Table — (DOCX) [file pone.0140341.s001.docx]

**S1 Table.** PCR temperature profiles and mixtures.

| Fragment | PCR program |
| --- | --- |
| COIF/R | 96ºC/4min-(94ºC/30s-48ºC/30s-72ºC/1min)*45cycles-72ºC/7min^a^ |
| 16SarL/brH | 95ºC/15min-(94ºC/1min-46ºC/30s-72ºC/2min)*37cycles-72ºC/8min^b^ |
| H3F/R | 95ºC/15min-(94ºC/1min-46ºC/30s-72ºC/2min)*37cycles-72ºC/8min^b^ |
| 28SF/R | 96ºC/4min-(94ºC/30s-48ºC/30s-72ºC/1min)*45cycles-72ºC/7min^a^ |
| 18S1F/9R | 96ºC/4min-(94ºC/30s-48ºC/30s-72ºC/1min)*45cycles-72ºC/7min^a^ |
| 435F/1213R | 95ºC/15min-(94ºC/1min-46ºC/30s-72ºC/2min)*37cycles-72ºC/8min^a^ |

^a^ Mixture used for every PCR reaction: 18.25 µl ddH_2_O, 2.5 µl Reaction Buffer (10X), 1.25 µl MgCl_2_, 0.5 µl each primer (10 µM), 0.5 µl dNTP’s, 0.5 BioTaq^TM^ DNA Polymerase (Bioline), 1 µl DNA template

^b^ Mixture used for every PCR reaction: 19.75 µl ddH_2_O, 2.5 µl Reaction Buffer (10X), 0.5 µl dNTP’s, 0.5 µl each primer (10 µM), 0.25 Hotmaster Taq^TM^ DNA Polymerase (5 PRIME), 1 µl DNA template
