# Supplementary material for: Bone-Eating Worms Spread: Insights into Shallow-Water Osedax (Annelida, Siboglinidae) from Antarctic, Subantarctic, and Mediterranean Waters
Source: PLoS One. 2015 Nov 18;10(11):e0140341. doi: 10.1371/journal.pone.0140341 (PMC4651350; doi:10.1371/journal.pone.0140341)
Supplement: S2 Table — Bold indicates new sequences. aSequence obtained from O. deceptionensis_4; bSequence obtained from O. deceptionensis_19. cSequences obtained from O. deceptionensis_3–5, O. deceptionensis_9–10, and O. deceptionensis_18–19. See S3 Table for further details on specimen assignation. (DOCX) [file pone.0140341.s002.docx]

**S2 Table.** Taxa included in the molecular phylogenetic analyses (*Osedax* and *Osedax* endosymbionts) with NCBI GenBank accession numbers. Bold indicates new sequences. ^a^Sequence obtained from *O. deceptionensis*_4; ^b^Sequence obtained from *O. deceptionensis*_19. ^c^Sequences obtained from *O. deceptionensis*_3–5, *O. deceptionensis*_9–10, and *O. deceptionensis*_18–19. See S3 Table for further details on specimen assignation.

| **Terminal taxa** | ***COI*** | ***16S*** | ***18S*** | ***28S*** | ***H3*** |
| --- | --- | --- | --- | --- | --- |
| *Cirratulus cirratus* (Müller, 1776) | GU672480 | DQ779609 | DQ779645 | DQ779683 | DQ779724 |
| *Lamellibrachia columna* Webb, 1969 | DQ996645 | FJ347646 | FJ347679 | --- | FJ347696 |
| *Oligobrachia haakonmosbiensis* Smirnov, 2000 | FM178481 | --- | AM883186 | --- | --- |
| *Riftia pachyptila* Jones, 1985 | KP119562 | KP119573 | KP119591 | KP119582 | KP119555 |
| *Sclerolinum brattstromi* Webb, 1964 | FJ347644 | FJ347645 | FJ347680 | FJ347677 | FJ347697 |
| *Sclerolinum contortum* Smirnov, 2000 | FM178480 | --- | AM883187 | --- | --- |
| *Siboglinum fiordicum* Webb, 1963 | --- | AF315039 | AF315060 | --- | DQ77976 |
| *Osedax* |  |  |  |  |  |
| *O. priapus* Rouse et al., 2015 | KP119564 | KP119579 | KP119595 | KP119586 | KP119556 |
| *O. rubiplumus* Rouse et al., 2004 | EU852488 | FJ347656 | FJ347681 | FJ347671 | FJ347704 |
| *O. frankpressi* Rouse et al., 2004 | FJ347607 | FJ347658 | FJ347682 | FJ347674 | FJ347705 |
| *O. mucofloris* Glover et al., 2005 | AY827562 | --- | AY941263 | --- | --- |
| *O. japonicus* Fujikura et a., 2006 | FM998111 | --- | FM995535 | --- | --- |
| *O. roseus* Rouse et al., 2008 | FJ347609 | FJ347657 | FJ347683 | FJ347670 | FJ347709 |
| *O. antarcticus* Glover et al., 2013 | KF444422 | KF444418 | KF444420 | --- | --- |
| *O. crouchi* Amon et al., 2014 | KJ598032 | KJ598035 | KJ598038 | --- | --- |
| *O. nordenskjoeldi* Amon et al., 2014 | KJ598033 | KJ598036 | KJ598039 | --- | --- |
| *O. rogersi* Amon et al., 2014 | KJ598034 | KJ598037 | KJ598040 | --- | --- |
| *O.* ‘spiral’ Braby et al., 2007 | FJ347638 | FJ347647 | FJ347693 | FJ347676 | FJ347703 |
| *O.* ‘yellow-collar’ Braby et al., 2007 | EU223337 | FJ347660 | FJ347689 | FJ347672 | FJ347706 |
| *O.* ‘orange-collar’ Braby et al., 2007 | FJ347629 | FJ347661 | FJ347690 | FJ347673 | FJ347707 |
| *O.* ‘nude-palp-A’ Jones et al., 2008 | FJ347624 | FJ347653 | FJ347687 | FJ347662 | FJ347702 |
| *O.* ‘nude-palp-B’ Jones et al., 2008 | EU236218 | FJ347652 | FJ347686 | FJ347665 | FJ347701 |
| *O.* ‘nude-palp-C’ Rouse et al., 2009 | FJ347626 | FJ347650 | FJ347688 | FJ347666 | FJ347710 |
| *O.* ‘nude-palp-D’ Vrijenhoek et al., 2009 | FJ347631 | FJ347649 | FJ347691 | FJ347667 | FJ347708 |
| *O.* ‘nude-palp-E’ Vrijenhoek et al., 2009 | FJ347635 | FJ347648 | FJ347692 | FJ347664 | FJ347700 |
| *O.* ‘nude-palp-F’ Vrijenhoek et al., 2009 | FJ347643 | FJ347651 | FJ347695 | FJ347663 | FJ347699 |
| *O.* ‘nude-palp-G’ Rouse et al., 2015 | KP119563 | KP119574 | KP119597 | KP119584 | KP119561 |
| *O.* ‘white-collar’ Vrijenhoek et al., 2009 | FJ347615 | FJ347659 | FJ347684 | FJ347675 | FJ347712 |
| *O.* ‘yellow-patch’ Vrijenhoek et al., 2009 | FJ347621 | FJ347654 | FJ347685 | FJ347668 | FJ347698 |
| *O.* ‘green-palp’ Vrijenhoek et al., 2009 | FJ347642 | FJ347655 | FJ347694 | FJ347669 | FJ347711 |
| *O.* ‘MB16’ Salathé & Vrijenhoek 2012 | JX280613 | KP119581 | KP119592 | KP119588 | KP119560 |
| *O.* ‘MB17’ Salathé & Vrijenhoek 2012 | JX280609 | KP119580 | KP119593 | KP119589 | KP119559 |
| *O. deceptionensis* Taboada et al., 2013 | **KT860545^a^** | KF444419 | **KT860543^a^** | **KT860544^b^** | **KT860546^a^** |
| *O.* ‘mediterranea’ This study | **KT860548** | **KT860551** | **KT860550** | **KT860549** | **KT860547** |
| *Osedax* endosymbionts |  |  |  |  |  |
| *O. deceptionensis* Oceanospirillales | --- | **KT873287–KT873293^c^** | --- | --- | --- |
| *O.* ‘mediterranea’ Oceanospirillales | --- | **KT873294** | --- | --- | --- |
