# Supplementary material for: Bone-Eating Worms Spread: Insights into Shallow-Water Osedax (Annelida, Siboglinidae) from Antarctic, Subantarctic, and Mediterranean Waters
Source: PLoS One. 2015 Nov 18;10(11):e0140341. doi: 10.1371/journal.pone.0140341 (PMC4651350; doi:10.1371/journal.pone.0140341)
Supplement: S3 Table — aSee Table 1 for further details. bSee Fig 8 (haplotype network) for further details. (DOCX) [file pone.0140341.s003.docx]

**S3 Table.** Information about the *COI* sequences used in the haplotype network, including the code of the individual, area and year of collection, haplotype assigned, NCBI GenBank accession number, and the part of the organism used for the DNA extraction. ^a^See Table 1 for further details. ^b^See Figure 8 (haplotype network) for further details.

| ***Osedax deceptionensis* code** | **Area of collection and Year^a^** | **Haplotype assigned^b^** | **NCBI accession number** | **Part used for DNA extraction** |
| --- | --- | --- | --- | --- |
| *O. deceptionensis*_2010 | Deception Is., 2010 | H9 | KT867774 | roots |
| *O. deceptionensis*_1 | Deception Is., 2013 | H6 | KT867775 | palps |
| *O. deceptionensis*_2 | Deception Is., 2013 | H6 | KT867776 | palps |
| *O. deceptionensis*_3 | Deception Is., 2013 | H10 | KT867777 | roots |
| *O. deceptionensis*_4 | Deception Is., 2013 | H11 | KT867790 | roots |
| *O. deceptionensis*_5 | Deception Is., 2013 | H3 | KT867778 | roots |
| *O. deceptionensis*_6 | Deception Is., 2013 | H6 | KT867779 | palps |
| *O. deceptionensis*_7 | Deception Is., 2013 | H1 | KT867793 | palps |
| *O. deceptionensis*_8 | Deception Is., 2013 | H6 | KT867780 | palps |
| *O. deceptionensis*_9 | Deception Is., 2013 | H2 | KT867781 | roots |
| *O. deceptionensis*_10 | Deception Is., 2013 | H7 | KT867782 | roots |
| *O. deceptionensis*_11 | Deception Is., 2013 | H8 | KT867783 | palps |
| *O. deceptionensis*_12 | Deception Is., 2013 | H4 | KT867784 | palps |
| *O. deceptionensis*_13 | Deception Is., 2013 | H6 | KT867785 | palps |
| *O. deceptionensis*_14 | Deception Is., 2013 | H6 | KT867786 | palps |
| *O. deceptionensis*_15 | Deception Is., 2013 | H6 | KT867787 | palps |
| *O. deceptionensis*_16 | Deception Is., 2013 | H6 | KT867788 | roots |
| *O. deceptionensis*_17 | Deception Is., 2013 | H5 | KT867789 | roots |
| *O. deceptionensis*_18 | South Georgia Is., 2013 | H9 | KT867791 | roots |
| *O. deceptionensis*_19 | South Georgia Is., 2013 | H12 | KT867792 | roots |
