# Supplementary material for: Bone-Eating Worms Spread: Insights into Shallow-Water Osedax (Annelida, Siboglinidae) from Antarctic, Subantarctic, and Mediterranean Waters
Source: PLoS One. 2015 Nov 18;10(11):e0140341. doi: 10.1371/journal.pone.0140341 (PMC4651350; doi:10.1371/journal.pone.0140341)
Supplement: S4 Table — (DOCX) [file pone.0140341.s004.docx]

**S4 Table.** *COI* divergence values (*p-*distance) between *Osedax* species and OTUs.

|  | | *Osedax* species &  OTUs | | 1 | | 2 | | 3 | | 4 | | 5 | | 6 | | 7 | | 8 | | 9 | | 10 | | 11 | | 12 | | 13 | | 14 | | 15 | | 16 | | 17 | | 18 | | 19 | | 20 | | 21 | | 22 | | 23 | | 24 | | 25 | | 26 | | 27 | |  |  |
| --- | --- | --- | --- | --- | --- | --- | --- | --- | --- | --- | --- | --- | --- | --- | --- | --- | --- | --- | --- | --- | --- | --- | --- | --- | --- | --- | --- | --- | --- | --- | --- | --- | --- | --- | --- | --- | --- | --- | --- | --- | --- | --- | --- | --- | --- | --- | --- | --- | --- | --- | --- | --- | --- | --- | --- | --- | --- | --- | --- |
| 1 | | *O. japonicus* | | – | |  | |  | |  | |  | |  | |  | |  | |  | |  | |  | |  | |  | |  | |  | |  | |  | |  | |  | |  | |  | |  | |  | |  | |  | |  | |  | |  |  |
| 2 | | *O. antarcticus* | | 0.189 | | – | |  | |  | |  | |  | |  | |  | |  | |  | |  | |  | |  | |  | |  | |  | |  | |  | |  | |  | |  | |  | |  | |  | |  | |  | |  | |  |  |
| 3 | | *O. mucofloris* | | 0.153 | | 0.212 | | – | |  | |  | |  | |  | |  | |  | |  | |  | |  | |  | |  | |  | |  | |  | |  | |  | |  | |  | |  | |  | |  | |  | |  | |  | |  |  |
| 4 | | *O. rubiplumus* | | 0.167 | | 0.189 | | 0.206 | | – | |  | |  | |  | |  | |  | |  | |  | |  | |  | |  | |  | |  | |  | |  | |  | |  | |  | |  | |  | |  | |  | |  | |  | |  |  |
| 5 | | *O.* 'MB17' | | 0.186 | | 0.186 | | 0.206 | | 0.172 | | – | |  | |  | |  | |  | |  | |  | |  | |  | |  | |  | |  | |  | |  | |  | |  | |  | |  | |  | |  | |  | |  | |  | |  |  |
| 6 | | *O. frankpressi* | | 0.167 | | 0.189 | | 0.175 | | 0.161 | | 0.181 | | – | |  | |  | |  | |  | |  | |  | |  | |  | |  | |  | |  | |  | |  | |  | |  | |  | |  | |  | |  | |  | |  | |  |  |
| 7 | | *O. roseus* | | 0.153 | | 0.184 | | 0.181 | | 0.172 | | 0.201 | | 0.153 | | – | |  | |  | |  | |  | |  | |  | |  | |  | |  | |  | |  | |  | |  | |  | |  | |  | |  | |  | |  | |  | |  |  |
| 8 | | *O. '*white-collar' | | 0.164 | | 0.198 | | 0.181 | | 0.178 | | 0.201 | | 0.184 | | 0.178 | | – | |  | |  | |  | |  | |  | |  | |  | |  | |  | |  | |  | |  | |  | |  | |  | |  | |  | |  | |  | |  |  |
| 9 | | *O. '*nudepalp-A' | | 0.184 | | 0.186 | | 0.189 | | 0.195 | | 0.192 | | 0.178 | | 0.181 | | 0.209 | | – | |  | |  | |  | |  | |  | |  | |  | |  | |  | |  | |  | |  | |  | |  | |  | |  | |  | |  | |  |  |
| 10 | | *O. '*nudepalp-B' | | 0.184 | | 0.209 | | 0.189 | | 0.206 | | 0.229 | | 0.169 | | 0.184 | | 0.203 | | 0.153 | | – | |  | |  | |  | |  | |  | |  | |  | |  | |  | |  | |  | |  | |  | |  | |  | |  | |  | |  |  |
| 11 | | *O. '*nudepalp-C' | | 0.192 | | 0.189 | | 0.181 | | 0.198 | | 0.198 | | 0.172 | | 0.195 | | 0.209 | | 0.178 | | 0.141 | | – | |  | |  | |  | |  | |  | |  | |  | |  | |  | |  | |  | |  | |  | |  | |  | |  | |  |  |
| 12 | | *O. '*nudepalp-D' | | 0.201 | | 0.184 | | 0.195 | | 0.195 | | 0.192 | | 0.192 | | 0.195 | | 0.186 | | 0.175 | | 0.184 | | 0.172 | | – | |  | |  | |  | |  | |  | |  | |  | |  | |  | |  | |  | |  | |  | |  | |  | |  |  |
| 13 | | *O. '*nudepalp-E' | | 0.209 | | 0.186 | | 0.169 | | 0.220 | | 0.218 | | 0.198 | | 0.198 | | 0.206 | | 0.203 | | 0.164 | | 0.144 | | 0.158 | | – | |  | |  | |  | |  | |  | |  | |  | |  | |  | |  | |  | |  | |  | |  | |  |  |
| 14 | | *O. '*nudepalp-F' | | 0.181 | | 0.161 | | 0.198 | | 0.184 | | 0.161 | | 0.203 | | 0.189 | | 0.184 | | 0.209 | | 0.181 | | 0.155 | | 0.181 | | 0.181 | | – | |  | |  | |  | |  | |  | |  | |  | |  | |  | |  | |  | |  | |  | |  |  |
| 15 | | *O.* 'nudepalp-G' | | 0.169 | | 0.184 | | 0.186 | | 0.164 | | 0.218 | | 0.184 | | 0.206 | | 0.175 | | 0.175 | | 0.141 | | 0.147 | | 0.195 | | 0.181 | | 0.172 | | – | |  | |  | |  | |  | |  | |  | |  | |  | |  | |  | |  | |  | |  |  |
| 16 | | *O.* 'yellow-collar' | | 0.147 | | 0.218 | | 0.169 | | 0.169 | | 0.195 | | 0.153 | | 0.158 | | 0.172 | | 0.186 | | 0.198 | | 0.215 | | 0.201 | | 0.203 | | 0.195 | | 0.186 | | – | |  | |  | |  | |  | |  | |  | |  | |  | |  | |  | |  | |  |  |
| 17 | | *O. '*orange-collar' | | 0.127 | | 0.215 | | 0.164 | | 0.169 | | 0.184 | | 0.158 | | 0.164 | | 0.164 | | 0.181 | | 0.206 | | 0.203 | | 0.189 | | 0.201 | | 0.198 | | 0.195 | | 0.076 | | – | |  | |  | |  | |  | |  | |  | |  | |  | |  | |  | |  |  |
| 18 | | *O. spiral* | | 0.189 | | 0.212 | | 0.178 | | 0.220 | | 0.232 | | 0.184 | | 0.198 | | 0.178 | | 0.201 | | 0.158 | | 0.178 | | 0.167 | | 0.192 | | 0.192 | | 0.192 | | 0.218 | | 0.209 | | – | |  | |  | |  | |  | |  | |  | |  | |  | |  | |  |  |
| 19 | | O. 'MB16' | | 0.150 | | 0.201 | | 0.178 | | 0.178 | | 0.195 | | 0.178 | | 0.186 | | 0.065 | | 0.223 | | 0.198 | | 0.206 | | 0.198 | | 0.198 | | 0.175 | | 0.172 | | 0.178 | | 0.181 | | 0.186 | | – | |  | |  | |  | |  | |  | |  | |  | |  | |  |  |
| 20 | | *O. crouchi* | | 0.192 | | 0.155 | | 0.169 | | 0.189 | | 0.181 | | 0.169 | | 0.184 | | 0.181 | | 0.186 | | 0.169 | | 0.158 | | 0.184 | | 0.164 | | 0.150 | | 0.167 | | 0.189 | | 0.195 | | 0.189 | | 0.178 | | – | |  | |  | |  | |  | |  | |  | |  | |  |  |
| 21 | | *O. nordenskjoeldi* | | 0.178 | | 0.181 | | 0.186 | | 0.218 | | 0.186 | | 0.186 | | 0.192 | | 0.192 | | 0.153 | | 0.175 | | 0.186 | | 0.172 | | 0.169 | | 0.201 | | 0.164 | | 0.206 | | 0.198 | | 0.209 | | 0.181 | | 0.153 | | – | |  | |  | |  | |  | |  | |  | |  |  |
| 22 | | *O. rogersi* | | 0.164 | | 0.164 | | 0.192 | | 0.201 | | 0.178 | | 0.192 | | 0.195 | | 0.178 | | 0.164 | | 0.169 | | 0.169 | | 0.167 | | 0.178 | | 0.150 | | 0.161 | | 0.178 | | 0.184 | | 0.189 | | 0.167 | | 0.164 | | 0.181 | | – | |  | |  | |  | |  | |  | |  |  |
| 23 | | *O. deceptionensis* | | 0.189 | | 0.184 | | 0.203 | | 0.206 | | 0.203 | | 0.192 | | 0.198 | | 0.240 | | 0.195 | | 0.198 | | 0.212 | | 0.198 | | 0.195 | | 0.201 | | 0.195 | | 0.203 | | 0.201 | | 0.218 | | 0.232 | | 0.201 | | 0.192 | | 0.209 | | – | |  | |  | |  | |  | |  |  |
| 24 | | *O. priapus* | | 0.178 | | 0.186 | | 0.215 | | 0.192 | | 0.212 | | 0.186 | | 0.181 | | 0.206 | | 0.172 | | 0.161 | | 0.181 | | 0.172 | | 0.181 | | 0.167 | | 0.155 | | 0.186 | | 0.206 | | 0.172 | | 0.192 | | 0.186 | | 0.169 | | 0.158 | | 0.206 | | – | |  | |  | |  | |  |  |
| 25 | | *O. '*yellow-patch' | | 0.175 | | 0.229 | | 0.184 | | 0.206 | | 0.206 | | 0.198 | | 0.209 | | 0.209 | | 0.209 | | 0.192 | | 0.206 | | 0.206 | | 0.178 | | 0.212 | | 0.192 | | 0.167 | | 0.169 | | 0.192 | | 0.201 | | 0.181 | | 0.192 | | 0.184 | | 0.198 | | 0.203 | | – | |  | |  | |  |  |
| 26 | | *O.'* green-palp' | | 0.186 | | 0.169 | | 0.201 | | 0.167 | | 0.175 | | 0.167 | | 0.175 | | 0.189 | | 0.153 | | 0.167 | | 0.181 | | 0.161 | | 0.172 | | 0.178 | | 0.164 | | 0.172 | | 0.172 | | 0.186 | | 0.201 | | 0.164 | | 0.164 | | 0.175 | | 0.186 | | 0.138 | | 0.186 | | – | |  | |  |  |
| 27 | | *O.*' mediterranea' | | 0.167 | | 0.203 | | 0.218 | | 0.212 | | 0.178 | | 0.178 | | 0.169 | | 0.172 | | 0.178 | | 0.186 | | 0.201 | | 0.198 | | 0.220 | | 0.195 | | 0.192 | | 0.192 | | 0.181 | | 0.184 | | 0.164 | | 0.181 | | 0.167 | | 0.175 | | 0.209 | | 0.147 | | 0.172 | | 0.155 | | – | | | |
