# Supplementary material for: Bone-Eating Worms Spread: Insights into Shallow-Water Osedax (Annelida, Siboglinidae) from Antarctic, Subantarctic, and Mediterranean Waters
Source: PLoS One. 2015 Nov 18;10(11):e0140341. doi: 10.1371/journal.pone.0140341 (PMC4651350; doi:10.1371/journal.pone.0140341)
Supplement: S5 Table — (DOCX) [file pone.0140341.s005.docx]

**S5 Table.** *COI* divergence values (Kimura 2 parameters) between *Osedax* species and OTUs.

|  | *Osedax* species &  OTUs | 1 | 2 | 3 | 4 | 5 | 6 | 7 | 8 | 9 | 10 | 11 | 12 | 13 | 14 | 15 | 16 | 17 | 18 | 19 | 20 | 21 | 22 | 23 | 24 | 25 | 26 | 27 |
| --- | --- | --- | --- | --- | --- | --- | --- | --- | --- | --- | --- | --- | --- | --- | --- | --- | --- | --- | --- | --- | --- | --- | --- | --- | --- | --- | --- | --- |
| 1 | *O. japonicus* | – |  |  |  |  |  |  |  |  |  |  |  |  |  |  |  |  |  |  |  |  |  |  |  |  |  |  |
| 2 | *O. antarcticus* | 0.254 | – |  |  |  |  |  |  |  |  |  |  |  |  |  |  |  |  |  |  |  |  |  |  |  |  |  |
| 3 | *O. mucofloris* | 0.192 | 0.297 | – |  |  |  |  |  |  |  |  |  |  |  |  |  |  |  |  |  |  |  |  |  |  |  |  |
| 4 | *O. rubiplumus* | 0.216 | 0.256 | 0.286 | – |  |  |  |  |  |  |  |  |  |  |  |  |  |  |  |  |  |  |  |  |  |  |  |
| 5 | *O.* 'MB17' | 0.250 | 0.251 | 0.285 | 0.228 | – |  |  |  |  |  |  |  |  |  |  |  |  |  |  |  |  |  |  |  |  |  |  |
| 6 | *O. frankpressi* | 0.215 | 0.254 | 0.229 | 0.205 | 0.239 | – |  |  |  |  |  |  |  |  |  |  |  |  |  |  |  |  |  |  |  |  |  |
| 7 | *O. roseus* | 0.192 | 0.246 | 0.241 | 0.225 | 0.276 | 0.193 | – |  |  |  |  |  |  |  |  |  |  |  |  |  |  |  |  |  |  |  |  |
| 8 | *O. '*white-collar' | 0.210 | 0.272 | 0.239 | 0.234 | 0.274 | 0.245 | 0.235 | – |  |  |  |  |  |  |  |  |  |  |  |  |  |  |  |  |  |  |  |
| 9 | *O. '*nudepalp-A' | 0.247 | 0.253 | 0.256 | 0.266 | 0.260 | 0.234 | 0.240 | 0.290 | – |  |  |  |  |  |  |  |  |  |  |  |  |  |  |  |  |  |  |
| 10 | *O. '*nudepalp-B' | 0.244 | 0.297 | 0.255 | 0.287 | 0.331 | 0.219 | 0.245 | 0.281 | 0.193 | – |  |  |  |  |  |  |  |  |  |  |  |  |  |  |  |  |  |
| 11 | *O. '*nudepalp-C' | 0.259 | 0.256 | 0.239 | 0.272 | 0.272 | 0.224 | 0.265 | 0.291 | 0.235 | 0.175 | – |  |  |  |  |  |  |  |  |  |  |  |  |  |  |  |  |
| 12 | *O. '*nudepalp-D' | 0.274 | 0.246 | 0.264 | 0.264 | 0.259 | 0.259 | 0.266 | 0.248 | 0.230 | 0.245 | 0.226 | – |  |  |  |  |  |  |  |  |  |  |  |  |  |  |  |
| 13 | *O. '*nudepalp-E' | 0.290 | 0.250 | 0.220 | 0.314 | 0.308 | 0.269 | 0.276 | 0.285 | 0.281 | 0.211 | 0.182 | 0.201 | – |  |  |  |  |  |  |  |  |  |  |  |  |  |  |
| 14 | *O. '*nudepalp-F' | 0.239 | 0.206 | 0.270 | 0.244 | 0.207 | 0.281 | 0.255 | 0.244 | 0.292 | 0.239 | 0.199 | 0.239 | 0.242 | – |  |  |  |  |  |  |  |  |  |  |  |  |  |
| 15 | *O.* 'nudepalp-G' | 0.220 | 0.248 | 0.249 | 0.211 | 0.310 | 0.243 | 0.285 | 0.230 | 0.229 | 0.175 | 0.183 | 0.264 | 0.239 | 0.226 | – |  |  |  |  |  |  |  |  |  |  |  |  |
| 16 | *O.* 'yellow-collar' | 0.187 | 0.308 | 0.221 | 0.221 | 0.266 | 0.192 | 0.203 | 0.225 | 0.248 | 0.270 | 0.304 | 0.274 | 0.283 | 0.266 | 0.248 | – |  |  |  |  |  |  |  |  |  |  |  |
| 17 | *O. '*orange-collar' | 0.154 | 0.301 | 0.212 | 0.221 | 0.245 | 0.201 | 0.212 | 0.210 | 0.239 | 0.285 | 0.280 | 0.253 | 0.276 | 0.270 | 0.264 | 0.088 | – |  |  |  |  |  |  |  |  |  |  |
| 18 | *O. spiral* | 0.253 | 0.298 | 0.234 | 0.312 | 0.337 | 0.245 | 0.272 | 0.234 | 0.275 | 0.203 | 0.234 | 0.215 | 0.261 | 0.258 | 0.260 | 0.309 | 0.291 | – |  |  |  |  |  |  |  |  |  |
| 19 | O. 'MB16' | 0.188 | 0.277 | 0.234 | 0.235 | 0.264 | 0.235 | 0.250 | 0.073 | 0.319 | 0.269 | 0.285 | 0.269 | 0.269 | 0.229 | 0.225 | 0.235 | 0.240 | 0.249 | – |  |  |  |  |  |  |  |  |
| 20 | *O. crouchi* | 0.259 | 0.199 | 0.221 | 0.254 | 0.240 | 0.220 | 0.247 | 0.239 | 0.252 | 0.223 | 0.202 | 0.243 | 0.213 | 0.187 | 0.217 | 0.254 | 0.264 | 0.256 | 0.234 | – |  |  |  |  |  |  |  |
| 21 | *O. nordenskjoeldi* | 0.233 | 0.244 | 0.249 | 0.308 | 0.251 | 0.249 | 0.261 | 0.259 | 0.192 | 0.229 | 0.249 | 0.225 | 0.220 | 0.275 | 0.210 | 0.285 | 0.270 | 0.290 | 0.239 | 0.195 | – |  |  |  |  |  |  |
| 22 | *O. rogersi* | 0.212 | 0.211 | 0.260 | 0.274 | 0.235 | 0.259 | 0.265 | 0.235 | 0.210 | 0.219 | 0.222 | 0.214 | 0.236 | 0.189 | 0.205 | 0.234 | 0.243 | 0.254 | 0.216 | 0.210 | 0.239 | – |  |  |  |  |  |
| 23 | *O. deceptionensis* | 0.253 | 0.247 | 0.284 | 0.288 | 0.285 | 0.260 | 0.269 | 0.357 | 0.268 | 0.271 | 0.301 | 0.271 | 0.266 | 0.278 | 0.266 | 0.281 | 0.276 | 0.310 | 0.340 | 0.280 | 0.263 | 0.293 | – |  |  |  |  |
| 24 | *O. priapus* | 0.235 | 0.256 | 0.302 | 0.261 | 0.299 | 0.248 | 0.240 | 0.287 | 0.228 | 0.208 | 0.241 | 0.228 | 0.242 | 0.215 | 0.199 | 0.248 | 0.286 | 0.225 | 0.260 | 0.253 | 0.225 | 0.201 | 0.292 | – |  |  |  |
| 25 | *O. '*yellow-patch' | 0.232 | 0.332 | 0.245 | 0.285 | 0.286 | 0.271 | 0.295 | 0.291 | 0.294 | 0.261 | 0.288 | 0.285 | 0.238 | 0.297 | 0.261 | 0.219 | 0.225 | 0.261 | 0.275 | 0.239 | 0.260 | 0.244 | 0.277 | 0.285 | – |  |  |
| 26 | *O.'* green-palp' | 0.248 | 0.222 | 0.275 | 0.214 | 0.230 | 0.214 | 0.229 | 0.254 | 0.193 | 0.217 | 0.241 | 0.205 | 0.227 | 0.234 | 0.211 | 0.224 | 0.224 | 0.249 | 0.275 | 0.214 | 0.214 | 0.229 | 0.251 | 0.171 | 0.251 | – |  |
| 27 | *O.*' mediterranea' | 0.214 | 0.286 | 0.308 | 0.296 | 0.239 | 0.235 | 0.219 | 0.225 | 0.235 | 0.249 | 0.275 | 0.271 | 0.317 | 0.265 | 0.258 | 0.259 | 0.239 | 0.244 | 0.211 | 0.240 | 0.217 | 0.230 | 0.296 | 0.183 | 0.225 | 0.197 | – |
