# Supplementary material for: Bone-Eating Worms Spread: Insights into Shallow-Water Osedax (Annelida, Siboglinidae) from Antarctic, Subantarctic, and Mediterranean Waters
Source: PLoS One. 2015 Nov 18;10(11):e0140341. doi: 10.1371/journal.pone.0140341 (PMC4651350; doi:10.1371/journal.pone.0140341)
Supplement: S6 Table — *See S3 Table for further details. (DOCX) [file pone.0140341.s006.docx]

**S6 Table.** *COI* divergence values (*p-*distance) between individuals of *Osedax deceptionensis*. *See S3 Table for further details.

|  | ***Osedax deceptionensis* code*** | **1** | **2** | **3** | **4** | **5** | **6** | **7** | **8** | **9** | **10** | **11** | **12** | **13** | **14** | **15** | **16** | **17** | **18** | **19** | **20** |
| --- | --- | --- | --- | --- | --- | --- | --- | --- | --- | --- | --- | --- | --- | --- | --- | --- | --- | --- | --- | --- | --- |
| 1 | *O. deceptionensis*_7 | – |  |  |  |  |  |  |  |  |  |  |  |  |  |  |  |  |  |  |  |
| 2 | *O. deceptionensis*_9 | 0.037 | – |  |  |  |  |  |  |  |  |  |  |  |  |  |  |  |  |  |  |
| 3 | *O. deceptionensis*_5 | 0.029 | 0.016 | – |  |  |  |  |  |  |  |  |  |  |  |  |  |  |  |  |  |
| 4 | *O. deceptionensis*_12 | 0.034 | 0.018 | 0.008 | – |  |  |  |  |  |  |  |  |  |  |  |  |  |  |  |  |
| 5 | *O. deceptionensis*_17 | 0.032 | 0.016 | 0.005 | 0.008 | – |  |  |  |  |  |  |  |  |  |  |  |  |  |  |  |
| 6 | *O. deceptionensis*_15 | 0.029 | 0.013 | 0.003 | 0.005 | 0.003 | – |  |  |  |  |  |  |  |  |  |  |  |  |  |  |
| 7 | *O. deceptionensis*_6 | 0.029 | 0.013 | 0.003 | 0.005 | 0.003 | 0.000 | – |  |  |  |  |  |  |  |  |  |  |  |  |  |
| 8 | *O. deceptionensis*_8 | 0.029 | 0.013 | 0.003 | 0.005 | 0.003 | 0.000 | 0.000 | – |  |  |  |  |  |  |  |  |  |  |  |  |
| 9 | *O. deceptionensis*_13 | 0.029 | 0.013 | 0.003 | 0.005 | 0.003 | 0.000 | 0.000 | 0.000 | – |  |  |  |  |  |  |  |  |  |  |  |
| 10 | *O. deceptionensis*_14 | 0.029 | 0.013 | 0.003 | 0.005 | 0.003 | 0.000 | 0.000 | 0.000 | 0.000 | – |  |  |  |  |  |  |  |  |  |  |
| 11 | *O. deceptionensis*_16 | 0.029 | 0.013 | 0.003 | 0.005 | 0.003 | 0.000 | 0.000 | 0.000 | 0.000 | 0.000 | – |  |  |  |  |  |  |  |  |  |
| 12 | *O. deceptionensis*_10 | 0.034 | 0.024 | 0.013 | 0.016 | 0.013 | 0.011 | 0.011 | 0.011 | 0.011 | 0.011 | 0.011 | – |  |  |  |  |  |  |  |  |
| 13 | *O. deceptionensis*_11 | 0.034 | 0.029 | 0.018 | 0.021 | 0.018 | 0.016 | 0.016 | 0.016 | 0.016 | 0.016 | 0.016 | 0.011 | – |  |  |  |  |  |  |  |
| 14 | *O. deceptionensis*_2010 | 0.034 | 0.018 | 0.008 | 0.011 | 0.008 | 0.005 | 0.005 | 0.005 | 0.005 | 0.005 | 0.005 | 0.016 | 0.021 | – |  |  |  |  |  |  |
| 15 | *O. deceptionensis*_3 | 0.032 | 0.016 | 0.005 | 0.008 | 0.005 | 0.003 | 0.003 | 0.003 | 0.003 | 0.003 | 0.003 | 0.013 | 0.018 | 0.003 | – |  |  |  |  |  |
| 16 | *O. deceptionensis*_4 | 0.040 | 0.013 | 0.018 | 0.021 | 0.018 | 0.016 | 0.016 | 0.016 | 0.016 | 0.016 | 0.016 | 0.026 | 0.032 | 0.016 | 0.013 | – |  |  |  |  |
| 17 | *O. deceptionensis*_1 | 0.029 | 0.013 | 0.003 | 0.005 | 0.003 | 0.000 | 0.000 | 0.000 | 0.000 | 0.000 | 0.000 | 0.011 | 0.016 | 0.005 | 0.003 | 0.016 | – |  |  |  |
| 18 | *O. deceptionensis*_2 | 0.029 | 0.013 | 0.003 | 0.005 | 0.003 | 0.000 | 0.000 | 0.000 | 0.000 | 0.000 | 0.000 | 0.011 | 0.016 | 0.005 | 0.003 | 0.016 | 0.000 | – |  |  |
| 19 | *O. deceptionensis*_19 | 0.037 | 0.021 | 0.011 | 0.013 | 0.011 | 0.008 | 0.008 | 0.008 | 0.008 | 0.008 | 0.008 | 0.018 | 0.024 | 0.003 | 0.005 | 0.018 | 0.008 | 0.008 | – |  |
| 20 | *O. deceptionensis*_18 | 0.034 | 0.018 | 0.008 | 0.011 | 0.008 | 0.005 | 0.005 | 0.005 | 0.005 | 0.005 | 0.005 | 0.016 | 0.021 | 0.000 | 0.003 | 0.016 | 0.005 | 0.005 | 0.003 | – |
